# Mallet-Like Distal Phalanx Base Injuries in Children and Adolescents: A PRISMA-ScR Scoping Review and a Proposed, Unvalidated Phenotype-Based Assessment Framework

**DOI:** 10.3390/children13101290

**Published:** 2026-09-22

**Authors:** Simon Arvati, Sami Cherfan, Nolwen Lemonnier, Carmen-Mediana Ion, Francois Luc

**Affiliations:** 1 Department of Paediatric Orthopaedics, CHU Rouen, 1 Rue Germont, 76000 Rouen, France; carmen-mediana.ion@chu-rouen.fr; 2 Department of Orthopaedics and Traumatology, CHU Rouen, 1 Rue Germont, 76000 Rouen, France; sami.cherfan@chu-rouen.fr (S.C.); nolwen.lemonnier@chu-rouen.fr (N.L.)

**Keywords:** Seymour fracture, distal phalanx, pediatric hand, physeal injury, mallet finger, nail-bed injury, scoping review

## Abstract

Background/Objectives: A mallet-like posture in a child or adolescent can represent a closed tendon or dorsal avulsion injury, but it may instead signal a physeal or juxtaphyseal distal-phalanx fracture that communicates with the nail unit. We mapped the diagnostic and management evidence and developed a proposed phenotype-based assessment framework whose clinical performance has not been validated. Methods: Following Joanna Briggs Institute (JBI) guidance and the Preferred Reporting Items for Systematic Reviews and Meta-Analyses extension for Scoping Reviews (PRISMA-ScR), PubMed and OpenAlex were searched without date restrictions through 22 July 2026, with citation chasing and organizational-website searching. Two human reviewers independently screened titles/abstracts and assessed potentially eligible reports; disagreements were resolved by discussion and consensus. One reviewer charted standardized data and a second verified the displayed fields. Results: The historical search yielded 219 database occurrences and 161 unique records. After 84 title/abstract exclusions, 77 database reports were assessed; 26 were excluded and 51 included. Other methods contributed nine sources, yielding 60 included sources. Evidence was predominantly retrospective and heterogeneous. Immobilization duration was not comprehensively synthesized in this review; therefore, no universal duration is proposed. No diagnostic-accuracy study, formal certainty grade, universally supported antibiotic regimen, or validated fixation threshold was identified. Conclusions: Nail-unit findings can raise concern for communication and support urgent specialist assessment under local protocols. Closed, congruent, stable mallet injuries may be considered for splinting after a complete nail-unit examination. The framework is proposed and unvalidated.

## 1. Introduction

Seymour described a juxta-epiphyseal fracture of the terminal phalanx in children; later reports established the importance of nail-plate displacement, germinal-matrix interposition, nail-bed communication, and infection risk [1,2,3,4,5]. A visually similar distal interphalangeal (DIP) extension lag may instead reflect a closed tendinous mallet injury, a dorsal intra-articular avulsion, or another physeal or juxtaphyseal base injury [6,7,8,9]. The management consequence is therefore not the eponym itself but whether the injury communicates with the external environment and whether reduction and joint congruity are stable.

Terminology is inconsistent. An open physis denotes skeletal immaturity and remaining growth potential; it is not synonymous with an open fracture. Likewise, a radiographic Seymour variant describes morphology but cannot establish nail-bed communication, whereas ‘bony mallet’ defines a dorsal articular avulsion and does not establish wound status. Treating these labels as mutually exclusive diagnoses can either undertreat an open injury or overtreat a stable closed pattern.

Kiely et al. quantified infection-related associations in Seymour injuries in a systematic review and meta-analysis [10]. Ross et al. provided a recent clinical narrative review spanning recognition, differential diagnosis, management, and outcomes [5]. The present scoping review has a different purpose: it maps both open and closed mallet-like distal-phalanx phenotypes across five interacting dimensions and separates mapped source findings from an author-derived, unvalidated assessment scaffold.

This scoping review asked: in children and adolescents aged 18 years or younger; cohorts explicitly described as pediatric or adolescent; and sources addressing traumatic distal-phalanx base, physeal, juxtaphyseal, Seymour, or mallet-like injuries, how are phenotypes defined; which examination and imaging findings influence management; and what evidence informs antibiotics, irrigation/debridement, reduction, fixation, splinting, and follow-up? The secondary objective was to propose an explicitly unvalidated phenotype-based assessment framework. Age, cohort description, reported or imaged physeal status, and separability of mixed-age data are treated as distinct variables.

## 2. Materials and Methods

### 2.1. Design, Protocol, and Reporting

This scoping review followed Joanna Briggs Institute (JBI) methodological guidance and is reported according to the Preferred Reporting Items for Systematic Reviews and Meta-Analyses extension for Scoping Reviews (PRISMA-ScR) and the PRISMA extension for reporting literature searches (PRISMA-S) [11,12,13]. No prospective protocol registration or publicly accessible protocol was documented for this review. Eligibility, search logic, charting fields, and synthesis axes were fixed before the final reconciled screen and are preserved in Supplements S1 and S2.

### 2.2. Eligibility Criteria

Historical population eligibility was recorded as age 18 years or younger, an explicit pediatric/adolescent cohort description, or explicit skeletal immaturity. Pediatric-specific quantitative synthesis required separable pediatric data. Two legacy mixed-age reports with nonseparable or incompletely reported age strata were retained only as contextual phenotype or technique descriptions and were not used for pediatric-specific quantitative inference. Reported or imaged physeal status was charted when available and was not inferred from age alone. Because the 60-source map does not document skeletal immaturity for every source, the revised title uses the more accurate population label ‘Children and Adolescents’. No source was retrospectively removed. Concept: traumatic distal-phalanx physeal/juxtaphyseal injury; Seymour injury; communicating nail-unit injury; and pediatric bony or tendinous mallet injury relevant to differential diagnosis, stability, treatment, or outcome. Context: emergency, outpatient, operative, delayed, and infected care.

Adult-only injuries, toes/hallux, non-traumatic deformity, chronic reconstruction outside the acute question, protocols, preprints, conference abstracts, duplicate/reprinted reports, and broad hand publications without a separable target result were excluded. Professional or institutional guidance was used only for external clinical concordance and was not counted as included research evidence.

### 2.3. Information Sources and Search

PubMed and OpenAlex were searched without date restriction through 22 July 2026. Two PubMed title/abstract strategies retrieved 59 and 35 occurrences. An OpenAlex Works application programming interface (API) query using fulltext.search for the phrase “Seymour fracture” retrieved 114 occurrences; this field can match indexed titles, abstracts, and full-text content where available and is not a full-text-only search. A display_name.search query for “pediatric mallet” retrieved 11 occurrences. The broad OpenAlex query deliberately favored sensitivity and accepted low specificity during screening; it was not treated as equivalent to Embase. Backward and forward citation chasing and targeted professional/institutional website searches yielded 15 reports for separate assessment. Exact strings, endpoints, fields, pagination, timestamps, exports, and source accounting appear in Supplement S1.

Embase, Scopus, and Web of Science were not searched for the historical corpus, and no results from those databases are claimed in this review. PubMed, OpenAlex, citation chasing, and organizational searches provide complementary coverage but do not eliminate retrieval bias; the residual risk of missing eligible reports is retained as a limitation.

### 2.4. Deduplication and Source Selection

The four historical database searches produced 219 occurrences. Deterministic matching by digital object identifier (DOI), PubMed identifier (PMID), and normalized title plus year generated duplicate candidates; 10 occurrences were removed within PubMed, two within OpenAlex, and 46 across sources, leaving 161 unique database records. Two human reviewers independently screened titles/abstracts and assessed potentially eligible reports against the eligibility criteria; disagreements were resolved by discussion and consensus. The 161-record register contains one reconciled outcome per record; 77 database and 15 other-method reports were taken forward. Reasons for report-level exclusions are provided in Supplement S2.

### 2.5. Data Charting

A standardized charting form captured citation, origin, design and sample when reported, population or phenotype, the principal review-relevant contribution, one main methodological constraint, and explicitly indexed or extractable funding/support. Missing fields were not inferred, and absent indexed funding was not interpreted as no funding. High-stakes numerators, denominators, timing definitions, and source limitations were cross-checked against available reports. One reviewer populated the chart, and a second verified every displayed field and citation against the source report; discrepancies were resolved by consensus. No separate pilot log or formal agreement statistic was retained, and none was reconstructed.

### 2.6. Appraisal and Synthesis

Because this scoping review aimed to map rather than grade evidence, no study was excluded on quality grounds and no formal source-level critical appraisal or certainty grade was assigned. Design, directness, sample size, confounding by indication, treatment-selection bias, follow-up, and one principal source-specific limitation were described narratively. The synthesis was descriptive across five distinct but interacting dimensions: physeal maturity; morphology/location; nail-unit communication; DIP congruity/reduction stability; and timing/infection. Reviews and guidance were not counted as independent replication of primary cohorts. No new meta-analysis was performed.

### 2.7. Use of Artificial Intelligence Assisted Tools

OpenAI Codex and generative pre-trained transformer (GPT) assisted tools supported metadata normalization, duplicate-candidate identification, language editing, and document formatting during manuscript preparation and revision. These tools did not make final duplicate, eligibility, extraction, interpretive, or clinical decisions. Human reviewers completed source selection and charting verification, and the authors reviewed and edited all outputs and retain full responsibility for the manuscript.

## 3. Results

### 3.1. Source Selection

For the corpus frozen on 22 July 2026, 161 unique database records were screened and 84 were excluded at title/abstract. Of 77 database reports assessed, 26 were excluded and 51 included. Fifteen reports were assessed through other methods; six were excluded and nine included. The historical map therefore contains 60 sources (Figure 1), with a one-to-one included bibliography and source map in Supplement S2. An unchanged-string rerun on 20 August 2026 returned three OpenAlex candidates (W2951549029, W7202014356, and W7200484546). Francois Luc (F.L.) and Nolwen Lemonnier (N.L.) independently excluded all three, with consensus recorded on 25 August 2026. No source was added, and the historical PRISMA flow and n = 60 evidence map remain unchanged.

### 3.2. Characteristics and Structure of the Mapped Evidence

The map spans historical descriptions, retrospective cohorts, case series, distinct-phenotype case reports, technique studies, pediatric mallet comparators, clinical reviews, and systematic reviews [1,2,3,4,6,9,14,15,16,17,18,19,20,21,22,23,24,25,26,27,28,29,30,31,32]. Most primary evidence was retrospective, small, and subject to confounding by indication. Secondary reviews may reuse the same cohorts and were not interpreted as independent evidentiary units. No formal critical appraisal or certainty grading was performed. No study established the sensitivity, specificity, or likelihood ratios of a nail-fold sign or radiographic feature, and no prospective study validated a decision rule. The five interacting dimensions are summarized in Table 1.

### 3.3. Diagnostic Recognition

In a skeletally immature patient, proximal nail-plate displacement superficial to the eponychial fold; blood or laceration at the nail fold; or direct evidence of nail-bed injury raises concern for possible communication with a distal-phalanx physeal or juxtaphyseal fracture [5,7,8,33,34,35]. These findings are not validated diagnostic tests. Anteroposterior, oblique, and dedicated true lateral radiographs should be obtained, but equivocal imaging does not remove concern when nail-unit violation is suspected. Because diagnostic-accuracy estimates are unavailable, suspected or uncertain communication supports urgent specialist evaluation rather than a binary rule-out label.

### 3.4. Suspected Open Injury

The most consistent evidence signal concerns prompt, complete care of open Seymour injuries. Earlier antibiotics and debridement were associated with lower infection risk in observational cohorts and a meta-analysis [10,18,19,22,28]; these data do not prove causality. Suspected or uncertain communication supports prompt specialist assessment and management under the local pediatric open-fracture/hand-injury pathway. Depending on clinical and operative findings, care may include tetanus review, antibiotics, irrigation and tissue-sparing debridement, removal of interposed tissue, reduction, and nail-bed repair. The <24-h antibiotic and <48-h debridement categories used in observational studies are analytical definitions, not safe-delay windows, biological cutoffs, or universal regimens.

Published series described selected emergency-department pathways that combined analgesia or anesthesia, wound assessment, irrigation/debridement when indicated, nail-bed assessment, reduction, post-reduction imaging, immobilization, and follow-up [21,24,33]. These descriptions do not establish a validated or universally applicable emergency-department standard, and the reported components should not be converted into universal eligibility conditions. Setting and treatment decisions remain dependent on injury findings, specialist judgment, resources, and the local pathway.

### 3.5. Nail Plate and Fixation

The nail plate may need to be elevated or removed to visualize the fracture and free interposed germinal matrix. After nail-bed repair, routine replacement of the plate should not be presented as established for Seymour fractures. The Nail bed INJury Analysis (NINJA) trial is external contextual randomized evidence and is not included in the 60-source map [36]. It found no infection or cosmetic benefit from replacement in uncomplicated pediatric nail-bed repair, but infected injuries and distal-phalanx fractures requiring fixation were excluded. Its findings are therefore indirect for Seymour injuries; replacement or discard depends on operative findings, stabilization needs, and the local specialist protocol.

After wound care and removal of interposed tissue, selective Kirschner wire (K-wire) fixation may be considered when an acceptable reduction cannot be obtained or maintained or when early redisplacement occurs [21,23,24,25,32,37,38]. The mapped evidence does not establish a benefit for routine pinning. Observational comparisons are confounded by severity and co-interventions, and no universal displacement, articular-surface, or instability threshold has been validated for pediatric Seymour fractures. Reported associations between transphyseal K-wires and growth disturbance cannot be interpreted as causal.

### 3.6. Convincingly Closed Injuries

Only injuries judged closed after a complete nail-unit examination should be considered for a mallet pathway. For a congruent, stable closed bony or tendinous mallet injury, continuous DIP-extension splinting with the proximal interphalangeal joint free and early hand-therapy or specialist review are commonly reported [39,40,41,42,43]. Immobilization duration was not comprehensively synthesized in this review; therefore, no universal duration is proposed. Large fragments without volar subluxation may still be considered for splinting with radiographic confirmation of maintained congruity; volar subluxation, irreducibility, or loss of reduction supports surgical discussion. Absolute pediatric operative thresholds remain uncertain.

Operative series in selected child and adolescent mallet fractures reported favorable outcomes [44,45,46,47,48], but none establishes surgical superiority over a stable splint pathway; recent mini-reviews provide a very-low-certainty context rather than validation [49,50]. In the full text published by Yildiran et al., 20 surgically treated patients aged 4–17 years had zero residual extension lag and excellent Crawford results. No major complication, recurrence, pin-tract irritation, or temporary stiffness was observed in this selected series. The small mixed bony/tendinous cohort and absence of a comparator do not establish safety or surgical superiority. A closed distal-phalanx physeal or juxtaphyseal injury should not automatically enter a generic mallet pathway; specialist-selected immobilization is plausible only when communication has not been identified and reduction is stable, and direct evidence remains sparse [6,30,31,51,52].

### 3.7. Delayed/Infected Presentations and Surveillance

Delayed presentation with purulence, cellulitis, radiographic osteomyelitis, delayed or nonunion, or failed prior treatment supports urgent specialist assessment, deep cultures when clinically appropriate, and empiric then culture-directed management under local pediatric bone-and-joint-infection guidance [22,53,54,55]. A lower failure rate with clindamycin than cephalexin in one retrospective cohort should not be generalized across settings. No universal antibiotic agent, dose, route, or duration is supported for delayed or infected presentations; microbiology, bone involvement, prior therapy, and local guidance remain determinative.

Families should be counselled about infection, nail dystrophy, malunion or nonunion, recurrent deformity, stiffness, and possible physeal disturbance. Follow-up should document union, alignment, DIP motion, and nail growth, with longer surveillance when growth disturbance or nail deformity is suspected. Available studies cannot separate effects of the initial physeal injury and infection from those of treatment or transphyseal fixation; repeated reduction attempts and unnecessary physeal injury should be minimized. Figure 2 presents a proposed, unvalidated assessment scaffold. Table 2 summarizes illustrative evidence signals, and Table 3 summarizes cautious responses by coexisting dimension with the directness of the supporting evidence.

## 4. Discussion

### 4.1. Principal Findings

Mallet posture is a presentation, not a diagnosis. The mapped literature in children and adolescents supports decomposing the injury into distinct but interacting population/maturity, anatomical, wound, mechanical, and temporal dimensions. Nail-unit violation can outweigh subtle radiographs because the safety concern is possible communication with a physeal or juxtaphyseal fracture. Conversely, a genuinely closed, congruent, stable mallet injury need not receive open-fracture treatment solely because it occurs in a pediatric cohort.

### 4.2. Clinical Concordance

External standards agree with the safety direction, not the evidentiary strength or performance, of the proposed framework. British Society for Surgery of the Hand (BSSH) guidance supports radiographs, DIP-extension splinting for stable closed mallet injuries, same-day review for open injuries, and surgical discussion for subluxation [43,56]. Royal Children’s Hospital Melbourne (RCH) guidance treats Seymour injury as an open fracture requiring urgent hand-surgery input and local open-injury care [34,35]. These documents were not counted among the 60 mapped research sources. Their concordance is not independent validation: guidance may draw on the same primary literature and provides no sensitivity, specificity, safety, or calibration estimate for this framework.

### 4.3. Implications for Research

Prospective studies should standardize nail-unit examination, true-lateral adequacy, fracture morphology, open-injury definition, congruity and stability, treatment components, and minimum follow-up. Priority outcomes are diagnostic accuracy against operative findings, infection and osteomyelitis, maintained reduction, reoperation, union, nail growth, physeal disturbance, DIP motion, adherence, patient-reported function, and return to activity. The proposed framework requires prospective testing of interobserver reliability, safety, discrimination, and calibration before implementation as a rule.

### 4.4. Limitations

This review has limitations at each stage. Search coverage was restricted to PubMed and OpenAlex plus citation and organizational searching; absence of Embase, Scopus, and Web of Science may have left eligible reports undiscovered. OpenAlex is dynamic, and its fulltext.search field is not full-text-only. Three records newly returned on 20 August were independently excluded by F.L. and N.L. and did not alter the historical map. No database-language filter was applied, and encountered languages and translation procedures were not systematically logged; no unrestricted-language claim is therefore made. No prospective protocol registration or publicly accessible protocol was documented. The retained screening and deduplication registers show reconciled outcomes but do not preserve reviewer-level votes, timestamps, or a formal agreement statistic. One reviewer charted, and a second verified displayed fields rather than performing independent dual extraction. No formal critical appraisal or certainty grading was performed. Source types were heterogeneous, reviews may have reused primary cohorts, and most treatment evidence was retrospective and confounded by indication. Two legacy mixed-age reports with inseparable age strata were retained only for contextual description and not for pediatric-specific quantitative inference. Skeletal immaturity was not reported for every source; the title therefore uses the broader population label ‘Children and Adolescents’. Finally, no diagnostic-accuracy or prospective validation study was found, so the proposed framework is not a validated diagnostic or treatment rule.

## 5. Conclusions

In a child or adolescent with a mallet-like distal-phalanx injury, separately documenting reported maturity, morphology, nail-unit findings, DIP congruity/reduction stability, and timing/infection may reduce premature eponym-based classification. Suspected or uncertain communication supports urgent specialist evaluation under the local pediatric open-fracture/hand-injury pathway, even when radiographs are subtle. When communication has not been identified after complete examination and the joint is congruent and stable, a specialist-supervised splint pathway may be considered. Immobilization duration was not comprehensively synthesized in this review; therefore, no universal duration is proposed. Fixation decisions remain individualized because no universal numerical threshold is validated. The proposed framework structures assessment; it does not replace clinical judgment, local protocols, or prospective validation.

## Figures and Tables

**Figure 1:**
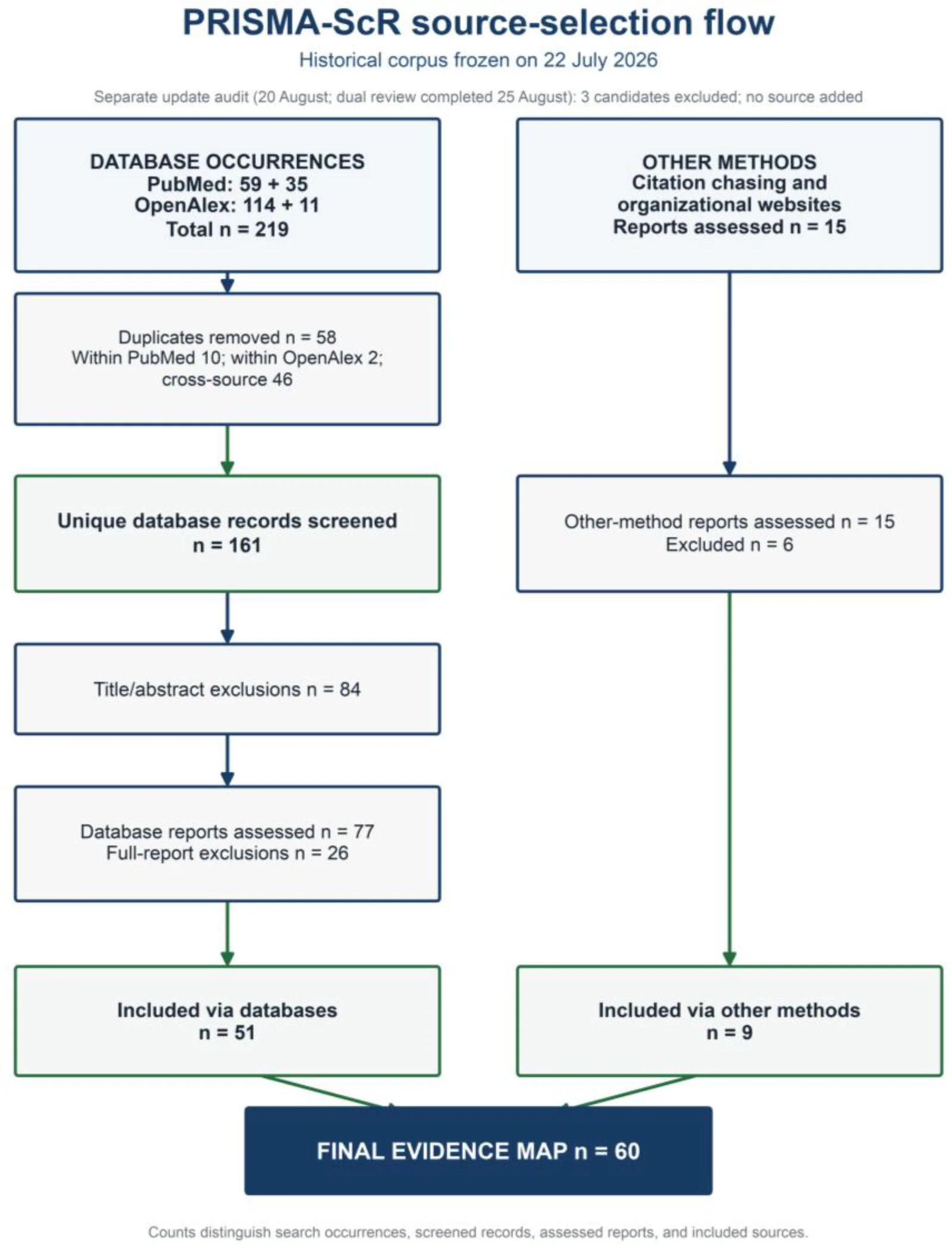
Preferred Reporting Items for Systematic Reviews and Meta-Analyses extension for Scoping Reviews (PRISMA-ScR) source-selection flow for the historical corpus frozen on 22 July 2026. Database counts distinguish occurrences from unique records; other-method reports are shown separately. The unchanged-string update check returned three new OpenAlex records; Francois Luc (F.L.) and Nolwen Lemonnier (N.L.) independently excluded all three on 25 August 2026. No source was added, and the historical flow is unchanged.

**Figure 2:**
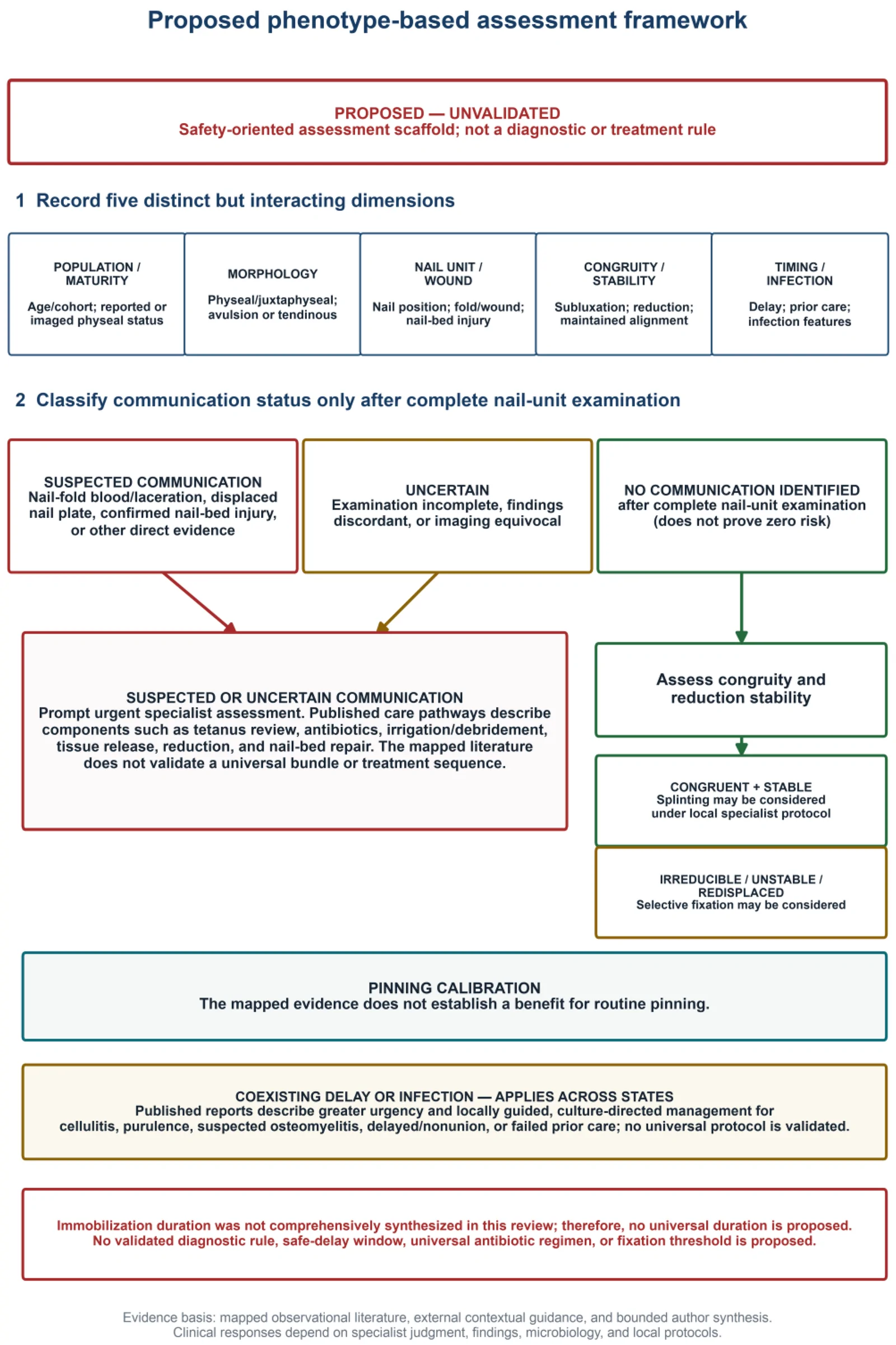
Proposed, unvalidated phenotype-based assessment framework. Suspected and uncertain communication converge on urgent specialist evaluation; absence of identified communication after complete examination does not exclude occult communication. The diagram is a deterministic vector schematic, not a diagnostic or treatment rule. Blue identifies assessment dimensions; red marks suspected communication, the urgent assessment branch, and caution statements; amber marks uncertainty, mechanical instability, or the delay/infection overlay; green marks the branch with no communication identified and the congruity/stability assessment. Teal identifies the separate pinning-evidence note. Colors organize the schematic and do not indicate validated risk categories.

**Table 1:** Five distinct but interacting population/maturity and injury dimensions to document before assigning an eponym or considering a management pathway. DIP, distal interphalangeal.

Dimension	Record Explicitly	Interpretive Consequence
Population/physeal maturity	Age/cohort descriptor; open, closing, or closed distal-phalanx physis only when reported or imaged	An open physis indicates skeletal immaturity and growth potential; age alone does not establish physeal status or wound openness.
Morphology/location	Physeal, juxtaphyseal, dorsal intra-articular avulsion, or no fracture	Separates extra-articular base injury from bony/tendinous mallet patterns; morphology alone does not establish communication.
Nail unit/wound	Nail-plate position; blood or laceration at the fold; nail-bed injury; and contamination	Raises concern for possible communication and supports urgent specialist evaluation; no individual sign is diagnostically validated.
Congruity/stability	DIP subluxation, displacement, reducibility, and maintained reduction	Informs whether specialist-selected immobilization is plausible or fixation should be discussed; no universal threshold is validated.
Timing/infection	Delay, prior care, cellulitis, purulence, osteomyelitis, and union	Modifies urgency, cultures, antimicrobial strategy, surgery, and surveillance; observed time categories are not safe-delay cutoffs.

**Table 2:** Eight illustrative evidence signals and interpretive constraints. The rows were selected post hoc as non-ranked examples based on direct relevance, sample size where informative, uniqueness of the signal, and relevance to high-stakes diagnostic or management decisions. NINJA is external contextual randomized evidence and is outside the 60-source map. NINJA, Nail bed INJury Analysis; RR, risk ratio; ED, emergency department.

Source	Design/Sample	Signal Used	Constraint
Kiely et al. 2022 [10]	Systematic review/meta-analysis; 10 non-randomized studies, 352 patients/355 fractures	Earlier debridement (RR 0.28), antibiotics within 24 h (RR 0.21), and both (RR 0.30) were associated with lower infection risk.	Heterogeneous observational evidence; <24 h and <48 h are analytical windows, not biological or safe-delay limits.
Reyes and Ho, 2017 [18]	Retrospective cohort; 34 patients/35 open Seymour fractures	Infection occurred in 0/11 acute-complete, 2/13 acute-incomplete, and 5/11 delayed-treatment injuries.	Small groups; delay, wound severity, and treatment completeness are intertwined.
Rask et al. 2021 [19]	Retrospective cohort; 52 patients/54 fractures; antibiotic timing reported for 46 fractures	Infection occurred in 2/29 receiving antibiotics within 24 h versus 13/17 receiving them later.	Eight fractures are outside the reported timing comparison; non-random allocation and residual confounding preclude a causal cutoff.
Lin et al. 2019 [21]	Retrospective cohort; 65 acute fractures	Fifty-eight were initially managed in the emergency department; 4/58 later required unplanned operation, commonly after redisplacement.	A published multi-component ED pathway, not a validated or universally applicable ED standard or eligibility checklist.
Samade et al. 2021 [22]	Retrospective cohort; 73 delayed presentations	Deep infection predicted operative treatment; antibiotics were associated with healing; clindamycin had fewer failures than cephalexin.	Confounding by indication and wide uncertainty; no universal antibiotic agent or duration follows from this comparison.
Lin and Samora, 2018 [39]	Retrospective cohort; 94 patients/99 pediatric mallet injuries	Almost all were treated nonoperatively; delay and nonadherence were associated with worse extension lag and complications.	Closed-mallet evidence is not transportable to suspected communication. Immobilization duration was not comprehensively synthesized in this review; therefore, no universal duration is proposed.
Jain et al. 2023 (NINJA) [36]—external context; outside the 60-source map	Multicenter randomized trial; 451 children (224 nail discarded, 227 nail replaced)	Nail replacement did not improve infection or cosmetic outcomes in uncomplicated nail-bed repair.	External contextual randomized evidence, indirect for Seymour injury: infected injuries and distal-phalanx fractures requiring fixation were excluded.
Yildiran et al. 2026 [48]	Retrospective surgical series; 20 patients aged 4–17 years; 12 bony/8 tendinous	All had zero residual lag and excellent Crawford results. No major complication, recurrence, pin-tract irritation, or temporary stiffness was observed in this selected series.	No comparator; selected mixed cohort and categorical ceiling effects do not establish safety or surgical superiority.

**Table 3:** Proposed evidence-informed responses by coexisting clinical dimension (unvalidated). Responses are precautionary prompts, not validated decision rules, diagnostic rules, or universal treatment instructions. DIP, distal interphalangeal.

Coexisting Dimension	Observed Pattern	Proposed Response	Evidence Basis/Directness
Communication status	Suspected or uncertain after complete nail-unit assessment; fold blood/laceration, displaced plate, nail-bed injury, and discordant or incomplete examination	Urgent specialist evaluation under the local pediatric open-fracture/hand-injury pathway. Treatment components depend on wound, imaging, operative findings, and local protocol.	Precautionary inference from observational Seymour cohorts plus external guidance; no diagnostic-accuracy study.
Closed mallet morphology	No communication identified after complete assessment; congruent and stable DIP; bony or tendinous mallet pattern	A specialist-supervised DIP-extension splint pathway may be considered, with radiographic confirmation and follow-up. Immobilization duration was not comprehensively synthesized in this review; therefore, no universal duration is proposed.	Retrospective pediatric mallet evidence plus external guidance; indirect if wound status is uncertain.
Closed physeal/juxtaphyseal morphology	No communication identified and reduction is acceptable and stable	Specialist-selected immobilization may be considered; do not default automatically to a generic mallet pathway.	Sparse observational/case evidence plus author precaution; direct comparative evidence is limited.
Mechanical instability	Irreducible, unstable, early redisplacement, or interposed germinal matrix/soft tissue	After wound assessment and tissue release when indicated, discuss selective fixation. No universal numerical threshold is supported.	Observational series with confounding by indication; no validated fixation threshold.
Delay or infection overlay	Cellulitis, purulence, suspected osteomyelitis, delayed/nonunion, or failed prior care; may coexist with any morphology	Increase urgency; obtain deep cultures when appropriate and use empiric then culture-directed management under local pediatric infection guidance.	Retrospective delayed-presentation cohorts and external guidance; no universal drug, dose, route, or duration.

## Data Availability

The original contributions presented in this study are included in the article and Supplementary Materials. Complete search strategies, dated source counts, eligibility decisions, and the included-source evidence map accompany the manuscript. Further inquiries can be directed to the corresponding authors.

## Supplementary Materials

The following supporting information can be downloaded at https://www.mdpi.com/article/10.3390/children13101290/s1. Supplement S1: historical search strategies, source accounting, reproducibility metadata, and the 20 August 2026 rerun with completed dual-reviewer decisions. Supplement S2: 60-source evidence map, included-source bibliography, reconciled database register, report-level exclusions, deduplication log, population/title audit, completed update-candidate decisions, and designation of NINJA as external contextual randomized evidence. PRISMA-ScR checklist: item-level section mapping.
