# Supplementary material for: Mallet-Like Distal Phalanx Base Injuries in Children and Adolescents: A PRISMA-ScR Scoping Review and a Proposed, Unvalidated Phenotype-Based Assessment Framework
Source: Children (Basel). 2026 Sep 22;13(10):1290. doi: 10.3390/children13101290 (PMC13703406; doi:10.3390/children13101290)
Supplement: Supplementary file 1 [file children-13-01290-s001.zip › children-4487609_PRISMA_ScR_Checklist.pdf]

COMPLETED REPORTING MAP

PRISMA-ScR Checklist

Mallet-Like Distal Phalanx Base Injuries in Children and Adolescents: A PRISMA-ScR Scoping Review and a Proposed, Unvalidated Phenotype-Based Assessment Framework

This checklist maps the revised manuscript by stable section identifier. Optional critical-appraisal items are not applicable because no formal appraisal was performed. Protocol, eligibility, search, selection, charting, funding, and disclosure items are reported in the manuscript and supplements.

Item	Topic	PRISMA-ScR requirement	Location	Status
1	Title	Identify the report as a scoping review.	Title; Methods §2.2; Supplement S2 §S2.8	Population label changed to ‘Children and Adolescents’; source-level classifications and the contextual-only role of two mixed-age reports are explicit in Supplement S2, and no source was retrospectively removed.
2	Structured summary	Background, objectives, eligibility, sources, selection/charting, numerical results, and conclusions.	Abstract	Reported; framework labelled proposed/unvalidated
3	Rationale	Explain the clinical uncertainty and why evidence mapping is appropriate.	Introduction §1	Reported
4	Objectives	State the Population-Concept-Context question and objectives.	Introduction §1, final paragraph	Reported
5	Protocol and registration	State whether a protocol exists and where it can be accessed.	Methods §2.1; Limitations §4.4	No prospective protocol registration or publicly accessible protocol was documented; reported in Methods §2.1 and Limitations §4.4.
6	Eligibility criteria	Specify population, concept, context, source types, dates, languages, and exclusions.	Methods §2.2; Supplement S1 §S1.1; Supplement S2 §S2.8	Population rules are explicit; no database-language filter was applied, and incomplete logging of encountered languages/translation is acknowledged.
7	Information sources	Name sources, interfaces, citation methods, and last search date.	Methods §2.3; Supplement S1 §§S1.2–S1.5 and S1.10	Historical search reported; three update candidates were independently excluded by FL and NL and did not change the corpus.
8	Search	Present reproducible strategies, limits, dates, counts, and deduplication.	Supplement S1 §§S1.2–S1.7 and S1.10–S1.11	Exact historical search fields/endpoints and completed update-candidate decisions are reported.
9	Selection of sources	Describe independent screening and consensus resolution.	Methods §2.4; Supplement S1 §S1.12; Supplement S2 §§S2.3–S2.4	Two reviewers independently screened records/reports with consensus resolution; reconciled outcomes and deterministic deduplication are documented.

Item	Topic	PRISMA-ScR requirement	Location	Status
10	Data charting process	Describe form development, extraction, verification, and consensus.	Methods §2.5; Supplement S1 §§S1.8 and S1.12	One reviewer charted and a second verified each displayed field and citation; no independent dual extraction or agreement statistic is claimed.
11	Data items	List all variables sought and assumptions/simplifications.	Methods §2.5; Supplement S2 §S2.1	Reported
12	Critical appraisal	If performed, explain the approach and its use.	Methods §2.6; Supplement S2 §S2.2	N/A optional item — no formal appraisal
13	Synthesis methods	Describe how charted data were grouped and summarized.	Methods §2.6	Reported
14	Selection results	Report all screening and eligibility counts with reasons.	Results §3.1; Figure 1; Supplement S1 §S1.10; Supplement S2 §S2.9	Historical flow retained after concordant exclusion of all three 20 August candidates.
15	Source characteristics	Present characteristics and citations for each included source.	Results §3.2; Supplement S2 §§S2.1, S2.5, and S2.8	Sixty historical sources mapped; the 'Children and Adolescents' title reflects the age/cohort-defined corpus without inferring physeal status.
16	Critical appraisal results	Present appraisal results when undertaken.	Methods §2.6; Supplement S2 §S2.2	N/A optional item — no appraisal results
17	Individual source results	Present relevant charted data for each source.	Results §§3.3–3.7; Tables 1–3; Supplement S2 §S2.1	Reported at descriptive charting level
18	Synthesis of results	Synthesize findings according to review objectives.	Results §§3.2–3.7; Tables 1–3; Figures 1–2	Reported with evidence-directness limits
19	Summary of evidence	Summarize main findings, uncertainty, and relevance.	Discussion §§4.1–4.2	Reported
20	Limitations	Report review-process and evidence limitations.	Discussion §4.4	Search, screening, charting, appraisal, source overlap, population/title, report-access, and validation limits reported.
21	Conclusions	Provide overall interpretation and implications.	Conclusion §5	Reported with conditional, unvalidated language
22	Funding	Describe review funding and source funding when reported.	Funding declaration; Supplement S2 §S2.1	Source funding/support is mapped where extractable; the review received no external funding.
