# Supplementary material for: Mallet-Like Distal Phalanx Base Injuries in Children and Adolescents: A PRISMA-ScR Scoping Review and a Proposed, Unvalidated Phenotype-Based Assessment Framework
Source: Children (Basel). 2026 Sep 22;13(10):1290. doi: 10.3390/children13101290 (PMC13703406; doi:10.3390/children13101290)
Supplement: Supplementary file 1 [file children-13-01290-s001.zip › children-4487609_Supplement_S1_Search_Strategies.pdf]

PRISMA-S

Supplement S1 — Complete Search Strategies

Mallet-Like Distal Phalanx Base Injuries in Children and Adolescents: A PRISMA-ScR Scoping Review and a Proposed, Unvalidated Phenotype-Based Assessment Framework

S1.1. Scope and date

The historical searches were run on 22 July 2026 without a date or database-language filter. Toe, toes, and hallux were excluded at title level because the review concerned fingers. 'Without date restriction' is used instead of 'from inception' because database coverage and indexing start dates differ. Encountered languages and translation procedures were not systematically logged; no unrestricted-language claim is made.

S1.2. PubMed query A — Seymour and distal-phalanx physeal injuries

("Seymour fracture"[Title/Abstract] OR "Seymour fractures"[Title/Abstract] OR (("distal phalanx"[Title/Abstract] OR "terminal phalanx"[Title/Abstract]) AND (physeal[Title/Abstract] OR physis[Title/Abstract] OR epiphyseal[Title/Abstract] OR juxtaepiphyseal[Title/Abstract] OR "juxta-epiphyseal"[Title/Abstract] OR juxtaphyseal[Title/Abstract] OR "juxta-physeal"[Title/Abstract]) AND (child*[Title/Abstract] OR pediatric*[Title/Abstract] OR paediatric*[Title/Abstract] OR adolescent*[Title/Abstract]))) NOT (toe[Title] OR toes[Title] OR hallux[Title])

Occurrences retrieved: 59. The submitted string above included toe[Title], toes[Title], and hallux[Title]. The archived July ESearch querytranslation omitted the separate toes[Title] term; the complete 59-ID export and translated query are retained without silently rewriting the submitted string.

S1.3. PubMed query B — pediatric mallet comparators

((("mallet finger"[Title/Abstract] OR "mallet fingers"[Title/Abstract] OR "mallet fracture"[Title/Abstract] OR "mallet fractures"[Title/Abstract]) AND (child*[Title/Abstract] OR pediatric*[Title/Abstract] OR paediatric*[Title/Abstract] OR adolescent*[Title/Abstract])) OR (("base of the distal phalanx"[Title/Abstract] OR "distal phalanx base"[Title/Abstract]) AND (child*[Title/Abstract] OR pediatric*[Title/Abstract] OR paediatric*[Title/Abstract] OR adolescent*[Title/Abstract]))) NOT (toe[Title] OR toes[Title] OR hallux[Title])

Occurrences retrieved: 35. The archived July export retained the submitted query and its translated query. On the 20 August rerun, PubMed flagged 'base of the distal phalanx' as a quoted phrase not found and translated that branch using 'distal phalanx base'; the rerun still returned 35 records. The raw warning, request URL, translation, and ID list are preserved with the update record.

S1.4. OpenAlex searches

OpenAlex Works API search A: GET /works?filter=fulltext.search:%22Seymour%20fracture%22&per_page=200. Historical occurrences retrieved: 114. The fulltext.search field can match indexed title, abstract, and full-text content where available; it is not exclusively a full-text field.

OpenAlex Works API search B: GET /works?filter=display_name.search:pediatric%20mallet&per_page=200. Historical occurrences retrieved: 11.

The Seymour phrase query was intentionally sensitive because terminology is inconsistent and relevant papers may not use the eponym in the title. This choice accepted low specificity and screened all 114 historical hits; it does not make OpenAlex equivalent to Embase, Scopus, or Web of Science. Archived records retain OpenAlex work ID, DOI, title, year, type, request path, selected fields, page-size metadata, and raw responses.

S1.5. Other methods

Backward reference lists and forward-citation links were checked from five prespecified seeds: Seymour 1966 (PMID 5939484), Ganayem 2005 (PMID 15958901), Lin and Samora 2018 (PMID 29776724), Kiely et al. 2022 (PMID 34810026), and Ross et al. 2026 (PMID 42434507). Publisher/PubMed reference links and OpenAlex work/citation pages were consulted on 22 July 2026. No exhaustive citation-index export was retained; the retained other-method decision register is therefore the complete record used for source accounting.

Targeted organizational searches were run on bssh.ac.uk and rch.org.au using the terms Seymour, mallet, open fracture, fingertip, nail injury, and phalangeal fracture. These standards were used only for external clinical concordance. Together, citation chasing and organizational searching yielded 15 unique reports assessed separately; nine research sources were included and six external, out-of-concept, preprint, or non-peer-reviewed documents were excluded from the 60-source map (Supplement S2.4).

S1.6. Deduplication

PubMed A+B produced 94 occurrences and 84 unique records (10 within-PubMed duplicates). OpenAlex A+B produced 125 occurrences and 123 unique works (two within-OpenAlex duplicates). Forty-six records overlapped PubMed and OpenAlex. Thus, 219 occurrences minus 58 duplicate occurrences yielded 161 unique database records. Matching order was PMID within PubMed, DOI when available, then normalized title plus year. The 58 removed occurrences and retained canonical records are preserved in the duplicate log.

S1.7. Source accounting

Source	Occurrences	Within-source unique	Final contribution
PubMed A	59	Part of 84 PubMed unique	Combined during cross-source deduplication
PubMed B	35	Part of 84 PubMed unique	Combined during cross-source deduplication
OpenAlex Seymour	114	Part of 123 OA unique	Combined during cross-source deduplication
OpenAlex pediatric mallet	11	Part of 123 OA unique	Combined during cross-source deduplication
Databases total	219	161	51 included
Other methods	15	15	9 included

S1.8. Selection and charting safeguards

- The retained 161-record register establishes one reconciled outcome per database record. Two human reviewers independently screened titles/abstracts and assessed potentially eligible reports; disagreements were resolved by discussion and consensus. Seventy-seven database and 15 other-method reports were taken forward.
- One reviewer charted standardized fields and a second verified every displayed field and citation against the source report; discrepancies were resolved by consensus. No separate pilot log, reviewer-level verification log, formal agreement statistic, or timestamped audit trail was retained.
- A peer-reviewed publication was required for inclusion; preprints, including the adolescent extension-block report, remained excluded.
- No separate pilot log for the charting form was retained and none was reconstructed.
- Search and selection counts were reconciled so that the 60 included source rows exactly match the included bibliography in Supplement S2.
- The 161-record register reports the reconciled consensus outcome. Reviewer-level votes, timestamps, and disagreement counts were not retained and were not reconstructed.
- OpenAI Codex and GPT-assisted tools supported metadata normalization, duplicate-candidate identification, language editing, and document formatting during manuscript preparation and revision. These tools did not make final duplicate, eligibility, extraction, interpretive, or clinical decisions. Human reviewers completed source selection and charting verification, and the authors reviewed and edited all outputs and retain full responsibility for the manuscript.

S1.9. Coverage limitation

Embase, Scopus, and Web of Science were not searched for the historical corpus, and no results from those databases are claimed in this review. PubMed, OpenAlex, citation chasing, and organizational searching provide complementary but incomplete coverage; residual retrieval bias remains possible.

S1.10. Unchanged-string rerun on 20 August 2026

The July strings were rerun unchanged through the official NCBI ESearch and OpenAlex Works API interfaces. Full request URLs, UTC retrieval timestamps, HTTP status, query translations, and raw JSON responses are preserved with the review records. The rerun was documented separately and did not alter the historical corpus. FL and NL independently assessed the three new OpenAlex records; all three were excluded, with consensus recorded on 25 August 2026. No source was added and the historical PRISMA flow remains unchanged.

Stream	22 Jul count	20 Aug count	Added IDs	Removed IDs
PubMed A	59	58	None	PMID 29667031 (historically excluded; index/query-translation drift)
PubMed B	35	35	None	None
OpenAlex fulltext.search	114	116	W2951549029; W7202014356	None
OpenAlex title search	11	12	W7200484546	None

S1.11. Update candidates — dual-reviewer decisions completed

OpenAlex ID	Title	FL/NL decision	Consensus stage/date
W2951549029	Interventions for treating fingertip entrapment injuries in children	EXCLUDE — both reviewers; broad fingertip publication without a separable target result.	REPORT_ASSESSMENT_EXCLUDE — 25 Aug 2026
W7202014356	Mallet Finger	EXCLUDE — both reviewers; submitted/unpublished repository item under the existing rule.	REPORT_ASSESSMENT_EXCLUDE — 25 Aug 2026
W7200484546	Physiotherapeutic intervention…Mallet Grading System	EXCLUDE — both reviewers; wrong concept and ineligible publication type.	TITLE_ABSTRACT_EXCLUDE — 25 Aug 2026

The controlling FL/NL register records concordant exclusion of all three candidates. W2951549029 and W7202014356 were excluded after report assessment; W7200484546 was excluded at title/abstract. No candidate entered the historical 60-source map, so no denominator changed.

S1.12. Human workflow and retained records

Stage	Documented process	Retained record / limitation
161-record title/abstract screen	Two human reviewers independently screened records; disagreements were resolved by discussion and consensus.	One reconciled final outcome per record; reviewer-level votes and timestamps were not retained.
77 database reports	Two reviewers assessed potentially eligible reports against the eligibility criteria, with consensus resolution.	51 inclusions and 26 exclusions with reasons; access level was not uniformly encoded in the retained register.
15 other-method reports	Reports identified through citation and organizational searching were assessed against the same criteria.	Nine inclusions and six exclusions; individual reviewer votes and dates were not retained.
58 duplicate removals	Deterministic and AI-assisted matching generated candidates; final duplicate decisions remained human.	Cluster/canonical-record log and deterministic matching basis are retained.
60-source charting	One reviewer charted standardized fields and a second verified every displayed field and citation.	No separate pilot log or formal agreement statistic was retained or reconstructed.
