# Supplementary material for: Mallet-Like Distal Phalanx Base Injuries in Children and Adolescents: A PRISMA-ScR Scoping Review and a Proposed, Unvalidated Phenotype-Based Assessment Framework
Source: Children (Basel). 2026 Sep 22;13(10):1290. doi: 10.3390/children13101290 (PMC13703406; doi:10.3390/children13101290)
Supplement: Supplementary file 1 [file children-13-01290-s001.zip › children-4487609_Supplement_S2_Evidence_Map.pdf]

PRISMA-ScR

Supplement S2 — Evidence Map and Eligibility Register

Mallet-Like Distal Phalanx Base Injuries in Children and Adolescents: A PRISMA-ScR Scoping Review and a Proposed, Unvalidated Phenotype-Based Assessment Framework

Historical invariant check (22 July 2026 freeze): 60 included source rows = 60 entries in the included bibliography = n=60 in Figure 1. Database sources: 51; other-method sources: 9. NINJA is external contextual randomized evidence and is not one of the 60. FL and NL independently excluded all three OpenAlex records newly returned on 20 August, with consensus on 25 August 2026; the historical denominator is unchanged.

S2.1. Charting fields and interpretation

The standardized map captured citation, origin, design/sample when reported, population or phenotype concept relevant to the review, the principal review-relevant contribution or result available from the report, one principal methodological constraint, and explicitly indexed or extractable funding/support. A field was not inferred when it was absent. Exact high-stakes numerical claims used in the synthesis are reproduced in manuscript Table 2 with denominators and interpretive constraints.

No.	ID	Year	Origin	Design/sample	Population/phenotype concept
1	PMID:5939484	1966	Other methods	Original clinical series, n=20 according to the full text; no PubMed abstract.	Foundational pediatric juxta-epiphyseal Seymour fracture and pseudo-mallet phenotype.
2	PMID:681400	1978	Other methods	Case report; sample details are not extractable from the PubMed record.	Traumatic nail avulsion with epiphyseal-plate injury and open-fracture communication.
3	PMID:488788	1979	Database	Observational series of 203 children with phalangeal fractures.	Broad pediatric phalangeal-fracture spectrum including distal-base epiphyseal injuries.
4	PMID:7364328	1980	Database	Case report, n=1 child; diagnosis was delayed by two weeks.	Dorsally displaced terminal epiphysis with attached extensor tendon, mimicking a mallet injury.
5	PMID:3408289	1988	Database	Case report, n=1; English abstract of a French-language article.	Open epiphyseal separation with nail avulsion complicated by flexor-tendon-sheath infection.
6	PMID:1624869	1992	Other methods	Case series, n=2.	Irreducible distal phalangeal epiphyseal injuries.
7	PMID:8349960	1993	Other methods	Case series, n=2 toddlers.	Occult distal-epiphysis fracture-dislocation after crush injury.
8	PMID:7797986	1995	Other methods	Clinical series of 19 children with 22 nail-bed injuries.	Pediatric nail-bed injuries; a Seymour-specific subgroup was not identified.
9	PMID:10357547	1999	Other methods	Case report, n=1.	Initially missed, angulated distal Salter-Harris II injury complicated by epiphyseal avascular necrosis.
10	PMID:11386767	2001	Other methods	Mixed child, adolescent, and adult case series, n=25; the pediatric subset is not separable.	Extra-articular transverse distal-phalanx-base fractures described as Seymour fractures.
11	PMID:15958901	2005	Database	Descriptive case series, n=7 children.	Juxta-epiphyseal Salter-Harris I/II or 1-2 mm distal-to-physis fracture presenting as a mallet mimic.

No.	ID	Year	Origin	Design/sample	Population/phenotype concept
12	PMID:18548355	2008	Database	Retrospective series, n=76 patients aged 1-17 years; 50 treated conservatively and 26 operatively.	Pediatric and adolescent mallet injuries classified using a modified Doyle system.
13	PMID:20724203	2010	Other methods	Case series, n=4; child/adult distribution is not extractable from the abstract.	Surf-associated Seymour-like juxta-epiphyseal fractures.
14	PMID:24206995	2013	Database	Clinical review; no primary patient sample.	Recognition and management of open Seymour fractures.
15	PMID:23351909	2013	Database	Retrospective series, n=24 skeletally immature patients; mean follow-up 10 years.	Seymour fracture with nail-bed injury and frequent nail subluxation.
16	PMID:24209954	2013	Other methods	Clinical review of pediatric hand fractures; no primary patient sample.	General pediatric hand-fracture context including Seymour and mallet injuries.
17	PMID:26279261	2015	Database	Case report, n=1 child.	Bony mallet avulsion without physeal injury.
18	DOI:10.7197/cmj.v37i2.5000079536	2015	Database	Single-center retrospective series, n=19 patients aged 9-16 years; 12 Doyle I and 7 Doyle IVa injuries.	Acute traumatic pediatric mallet injury treated conservatively.
19	PMID:27490217	2016	Database	Clinical review; no primary patient sample.	Problematic pediatric hand and wrist fractures, including Seymour and mallet patterns.
20	PMID:29786288	2016	Database	Surgical series, n=26 children/adolescents aged 1.1-17 years; mean follow-up 12.3 months.	Acute Seymour fractures.
21	PMID:28968312	2017	Database	Case report, n=1 female toddler.	Initially missed mallet injury in a toddler.
22	PMID:28735119	2017	Database	Retrospective series, n=13; mean age 10.3 years.	Open Seymour crush injuries caused by hoverboard misuse.
23	PMID:28465015	2017	Database	Retrospective cohort, n=206 pediatric distal-phalanx fractures; mean age 7.5 years.	Broad distal-phalanx cohort including 18% mallet and 6% Seymour fractures.
24	PMID:26327401	2017	Database	Retrospective cohort, 34 patients with 35 open Seymour fractures.	Salter-Harris I/II or juxta-epiphyseal fracture with nail-bed laceration.
25	PMID:29399000	2018	Database	Retrospective surgical series, n=17 adolescents; mean age 15.2 years; physes closed or nearly closed.	Bony mallet fracture with subluxation and/or at least one-third articular-surface involvement.
26	PMID:29776724	2018	Database	Retrospective cohort, 94 patients with 99 injured digits; mean age 13.7 years; 99% treated with an orthosis.	Pediatric mallet injury, 80% bony; effects of delay and adherence.
27	PMID:28719993	2018	Database	Comparative series, n=19: 11 Ishiguro fixation and 8 low-intensity pulsed ultrasound; allocation method not stated.	Displaced pediatric mallet fractures.
28	PMID:30358692	2019	Database	Retrospective cohort, n=65 acute fractures presenting within 24 hours in patients younger than 18 years.	Acute Seymour fracture and emergency-department management.
29	PMID:30730419	2019	Database	Retrospective surgical series, n=51: 38 acute and 13 chronic injuries; mean age 14.6 years.	Percutaneous reduction and pin fixation of pediatric mallet fractures.
30	PMID:33508522	2021	Database	Retrospective series: 21 operated patients, of whom only 12 were assessed at two years or later; mean age 9.3 years.	Unreduced Seymour fracture presenting after 24 hours and treated after debridement/repair without K-wire fixation.
31	PMID:30730385	2021	Database	Literature review covering 1985-2016; the number of included reports is not stated in the abstract; no primary patient sample.	Pediatric/adolescent upper-extremity casting and orthoses, including mallet finger.

No.	ID	Year	Origin	Design/sample	Population/phenotype concept
32	PMID:34842086	2021	Database	Retrospective acute Seymour series, n=29 patients; 24 operative, 5 closed reduction/splint, and 21 K-wire cases; mean age 7.9 years.	Acute Seymour management, antibiotics, and fixation.
33	PMID:32149995	2021	Database	Retrospective cohort, 52 patients with 54 fractures and more than 30 days of follow-up.	Seymour infection and antibiotic timing.
34	PMID:31597480	2021	Database	Single-institution retrospective cohort, n=73 delayed presentations beyond 24 hours; median delay 7 days.	Delayed or infected Seymour injury.
35	DOI:10.4103/atr.atr_9_22	2022	Database	Case report, n=1 six-year-old boy.	Seymour fracture with nail-bed interposition, nonunion, and nail deformity.
36	PMID:35703237	2022	Database	Retrospective single-center series, n=26, divided into three presentation/severity categories with category-specific treatment.	Typical acute, delayed-recognition, and crushed-open Seymour phenotypes.
37	PMID:36268384	2022	Database	Structured best-evidence topic; eight studies comprising five case series and three single-center retrospective cohorts.	K-wire fixation in pediatric Seymour fracture.
38	PMID:34810026	2022	Database	Systematic review and meta-analysis; 10 nonrandomized studies, 352 patients, and 355 fractures.	Pediatric Seymour debridement/antibiotic timing and complications.
39	PMID:35311365	2022	Database	Case report, n=1.	Displaced Salter-Harris III distal-phalanx juxtaphyseal variant mimicking mallet or Seymour injury.
40	PMID:37791208	2023	Database	Case report, n=1 patient with three simultaneous bony mallet fractures.	Multiple adolescent bony mallet injuries.
41	PMID:36607925	2023	Database	Retrospective comparative chart review, 66 operatively treated fractures: 36 classic and 30 radiographic variants.	Classic versus radiographic-variant Seymour phenotype.
42	PMID:36974281	2023	Database	Case report, n=1 13-year-old with a one-month delayed presentation.	Subacute, infected, unstable Seymour fracture.
43	PMID:37255640	2023	Database	Case report, n=1 with seven-year observation.	Late complication after adolescent mallet pin fixation.
44	DOI:10.1016/j.joscr.2023.09.007	2023	Database	Case series, n=2 boys aged 12 and 16 years.	Delayed-recognition Seymour fracture with acute osteomyelitis.
45	PMID:38561523	2024	Database	Retrospective cross-sectional long-term surgical series; 84 of 95 children included, mean age 14.8 years, mean follow-up 11.6 years.	Ishiguro extension-block K-wire treatment for pediatric bony mallet.
46	PMID:39354737	2024	Database	Clinical/narrative educational review in Swedish with an English abstract; no primary sample.	Recognition and initial management of Seymour fracture.
47	PMID:40863716	2025	Database	Case report, n=1 12-year-old girl.	Volar-displaced Salter-Harris III pediatric mallet fracture.
48	PMID:40687299	2025	Database	Systematic review with pooled descriptive/subgroup analyses; 24 studies and 915 open distal-phalanx fractures in adults and children.	Debridement timing, antibiotics, and complications of open distal-phalanx fractures.
49	PMID:40432862	2025	Database	Two-round Delphi consensus; 34 then 33 self-identified pediatric hand surgeons.	Pediatric fingertip treatment pathway including Seymour scenarios.
50	DOI:10.29245/2767-5130/2025/2.1221	2025	Database	Mini-review of studies published in 2014-2024; included-study count not reported; no primary sample.	Pediatric versus adult bony mallet management.

No.	ID	Year	Origin	Design/sample	Population/phenotype concept
51	DOI:10.1016/j.joscr.2024.12.001	2025	Database	Case series, n=2 teenagers.	Delayed Seymour fracture with pyogenic arthritis and osteomyelitis.
52	PMID:40861033	2025	Database	Surgical technique article; no comparative patient sample reported.	Operative Seymour management with nail-bed involvement.
53	DOI:10.47363/JSAR/2025(6)212	2025	Database	Review article using PubMed and Cochrane searches; included-study count not reported; no primary sample.	Pediatric bony mallet treatment and a proposed algorithm.
54	PMID:39603863	2025	Database	Retrospective comparative conservative cohort, n=31: 16 bony and 15 tendinous mallet injuries.	Acute pediatric bony versus tendinous mallet treated with a splint.
55	PMID:40235410	2025	Database	Retrospective acute Seymour series reported as n=28; mean follow-up 16.6 months.	Stable emergency-department management versus surgery after failed closed reduction.
56	PMID:41289484	2026	Database	Instructional Course Lecture/clinical review; no primary sample.	Pediatric physeal phalanx fractures, Seymour injury, and bony mallet indications.
57	PMID:41065608	2026	Database	Narrative clinical review; no primary sample.	Potentially problematic pediatric phalanx fractures.
58	PMID:42434507	2026	Database	Contemporary narrative clinical review; no primary sample.	Seymour definition, diagnosis, differential diagnosis, and acute/delayed management.
59	PMID:42058391	2026	Database	Narrative acute-care review supplemented by expert opinion; no primary sample.	Pediatric hand fractures/dislocations and referral.
60	DOI:10.4103/tjps.tjps_85_25	2026	Database	Retrospective single-center surgical series, n=20 patients aged 4–17 years; 12 bony and 8 tendinous injuries; mean follow-up 42.1 months.	Selected operative child/adolescent mallet cohort treated with a longitudinal K-wire.

S2.1 continued. Source-level contributions, constraints, and funding metadata

No.	ID	Review-relevant contribution/result	Principal constraint	Funding/support status
1	PMID:5939484	Conservative treatment was favored; among five pinned cases, one developed osteomyelitis requiring amputation and two had prolonged pin-track infections.	Small, descriptive, noncomparative 1966 series reflecting historical treatment and selection practices.	No funding statement indexed in the archived PubMed metadata; not interpreted as unfunded.
2	PMID:681400	No primary outcome is extractable; the report establishes the association between nail injury and an open physeal fracture.	No abstract; treatment and outcome details are not extractable from the archived metadata.	No funding statement indexed in the archived PubMed metadata; not interpreted as unfunded.
3	PMID:488788	Distal-phalanx-base epiphyseal fractures were reported to have often unsatisfactory outcomes.	The target subgroup size and outcomes are not separable from an anatomically heterogeneous cohort.	No funding statement indexed in the archived PubMed metadata; not interpreted as unfunded.
4	PMID:7364328	Open reduction was followed by satisfactory epiphyseal position and normal terminal-phalanx growth at three years.	Single case without a comparator.	No funding statement indexed in the archived PubMed metadata; not interpreted as unfunded.
5	PMID:3408289	Documents the risk of deep infection after an open distal physeal injury.	Patient, treatment, and final-outcome details are not extractable from the abstract.	No funding statement indexed in the archived PubMed metadata; not interpreted as unfunded.
6	PMID:1624869	In both cases, an eponychial-fold bridge prevented reduction.	Two cases; functional outcome and follow-up are not reported in the abstract.	No funding statement indexed in the archived PubMed metadata; not interpreted as unfunded.
7	PMID:8349960	Initially missed injuries presented years later with a dorsal mass, shortening, and DIP stiffness.	Two delayed presentations; no frequency estimate or treatment comparison.	No funding statement indexed in the archived PubMed metadata; not interpreted as unfunded.
8	PMID:7797986	After primary repair, normal nail growth was reported in all children and the overall result was rated good in 91%.	The population is broader than the target phenotype, with no separable Seymour outcome or comparator.	No funding statement indexed in the archived PubMed metadata; not interpreted as unfunded.
9	PMID:10357547	After open reduction and fixation, premature physeal closure and mild shortening occurred, but revascularization and good DIP function were reported.	The effects of the original injury and treatment cannot be separated.	No funding statement indexed in the archived PubMed metadata; not interpreted as unfunded.
10	PMID:11386767	Among 18 reduction-and-splint cases, one infection and three residual flexion deformities occurred; five K-wire cases healed without complication.	Mixed ages, nonrandom treatment allocation, and nonseparable pediatric results.	No funding statement indexed in the archived PubMed metadata; not interpreted as unfunded.
11	PMID:15958901	All underwent closed reduction; six received K-wires and one a splint, and all healed without complication.	Very small noncomparative series without a standardized functional measure.	No funding statement indexed in the archived PubMed metadata; not interpreted as unfunded.
12	PMID:18548355	No final extension deficit was seen in 81.5% overall and 94% of conservatively treated patients; six operative patients had poor outcomes, including two suture infections.	Heterogeneous lesions, strong confounding by indication, and nonrandom treatment comparison.	No funding statement indexed in the archived PubMed metadata; not interpreted as unfunded.
13	PMID:20724203	A 1.2-mm K-wire was used as a reduction joystick and then as fixation, followed by nail fixation.	Small mechanism-specific series; follow-up and complications are not reported in the abstract.	No funding statement indexed in the archived PubMed metadata; not interpreted as unfunded.
14	PMID:24206995	No primary outcome; narrative clinical context.	No abstract or new primary data; conclusions depend on narrative source selection.	No funding statement indexed in the archived PubMed metadata; not interpreted as unfunded.
15	PMID:23351909	Twenty-three of 24 regained full motion; no infection or flexion deformity was reported; minor physeal/nail irregularity occurred in five and isolated nail dystrophy in six.	Small, nonrandom treatment series with follow-up ranging from 1 to 18 years.	No funding statement indexed in the archived PubMed metadata; not interpreted as unfunded.

No.	ID	Review-relevant contribution/result	Principal constraint	Funding/support status
16	PMID:24209954	No primary outcome; narrative clinical context.	Broad review without new or separable target data.	Full-text acknowledgement reports NIAMS/NIH support including 2R01 AR047328-06 and K24 AR053120 (support attributed to an author; not necessarily direct study funding).
17	PMID:26279261	Open reduction and fixation were followed by union without complication.	Single rare case; exact age and follow-up are not extractable from the abstract.	No funding statement indexed in the archived PubMed metadata; not interpreted as unfunded.
18	DOI:10.7197/cmj.v37i2.5000079536	Splinting lasted four weeks for type I and five weeks for type IVa; all 19 had excellent Crawford outcomes, with two transient superficial skin problems.	Small selected cohort without a comparator; long-term and quantitative radiographic outcomes were not reported.	No PubMed funding field; no structured funding statement in the archived bibliographic record; not interpreted as unfunded.
19	PMID:27490217	No primary outcome; narrative clinical context.	Expert recommendations from a broad review without a direct treatment-effect estimate.	No funding statement indexed in the archived PubMed metadata; not interpreted as unfunded.
20	PMID:29786288	All fractures united and no infection occurred; one nail deformity and one residual 10-degree extension deficit were reported.	Single-center uncontrolled series with 2-24 months of follow-up and a uniform operative protocol.	No funding statement indexed in the archived PubMed metadata; not interpreted as unfunded.
21	PMID:28968312	Describes delayed diagnosis and subsequent treatment; the primary outcome is not extractable from the abstract.	Single case; precise treatment, final imaging, and functional outcome are absent from the abstract.	No funding statement indexed in the archived PubMed metadata; not interpreted as unfunded.
22	PMID:28735119	Describes a high-energy mechanism and a distribution mainly affecting middle and ring fingers of the nondominant hand.	Single-center, mechanism-specific series without treatment outcomes or follow-up in the abstract.	No funding statement indexed in the archived PubMed metadata; not interpreted as unfunded.
23	PMID:28465015	Complications occurred in 31% overall, 62% of Seymour fractures, and 49% of mallet fractures; infection, stiffness, and nail deformity predominated.	Code-based ascertainment and clinician-judged outcomes; subgroup treatments and detailed denominators are not extractable.	No funding statement indexed in the archived PubMed metadata; not interpreted as unfunded.
24	PMID:26327401	Infection occurred in 0/11 acute-complete, 2/13 acute-partial, and 5/11 delayed-treatment injuries; five of seven infections were osteomyelitis.	Small nonrandom groups in which delay, severity, and treatment completeness are intertwined.	No funding statement indexed in the archived PubMed metadata; not interpreted as unfunded.
25	PMID:29399000	Two major complications and two residual extension lags occurred, mainly after delay or nonadherence.	No nonoperative comparator, small sample, and limited applicability to open physes.	No funding statement indexed in the archived PubMed metadata; not interpreted as unfunded.
26	PMID:29776724	Mean final lag was 1 degree; lag occurred in 12% of acute versus 25% of delayed cases, and nonadherence was associated with 67% lag and 50% complications.	Noncausal associations with potential delay/adherence confounding and almost no surgical cases.	No funding statement indexed in the archived PubMed metadata; not interpreted as unfunded.
27	PMID:28719993	The ultrasound group had slower union but greater motion and 8/8 excellent outcomes; the pinned group had eight excellent and three good outcomes.	Very small nonrandom groups with incompletely described baseline differences, follow-up, and complications.	No funding statement indexed in the archived PubMed metadata; not interpreted as unfunded.
28	PMID:30358692	Fifty-eight were initially managed in the emergency department; four later required surgery, usually for redisplacement; complications were rare.	Nonrandom treatment selection; exact complication counts and the complete emergency-department protocol are not in the abstract.	No funding statement indexed in the archived PubMed metadata; not interpreted as unfunded.
29	PMID:30730419	All united; Crawford outcomes were excellent in 87% of acute and 77% of chronic cases; no infection, wire breakage, nail deformity, or unplanned surgery occurred.	No conservative comparator, operative selection, and mean follow-up of only 78.5 days.	No funding statement indexed in the archived PubMed metadata; not interpreted as unfunded.

No.	ID	Review-relevant contribution/result	Principal constraint	Funding/support status
30	PMID:33508522	One superficial infection resolved; final mean angulation was 0.50 degrees, length 98% of the opposite side, and ROM 99%.	Nine of 21 patients were not evaluated, the analyzed sample was small, and there was no pinned comparator; delays were 28-44 hours.	No funding statement indexed in the archived PubMed metadata; not interpreted as unfunded.
31	PMID:30730385	No primary outcome; provides narrative support for removable extension orthoses in nonsurgical mallet injury.	Broad non-Seymour review without a reported study count, formal appraisal, or pediatric mallet-specific pooled estimate.	No funding statement indexed in the archived PubMed metadata; not interpreted as unfunded.
32	PMID:34842086	At mean 10.8-month follow-up, final passive ROM and physeal arrest did not differ significantly by K-wire use; antibiotics were associated with lower infection risk (OR 0.04, 95% CI 0.006-0.2).	Small retrospective series with highly imbalanced selected treatment groups, co-interventions, and confounding by severity; association is not causal.	No funding statement indexed in the archived PubMed metadata; not interpreted as unfunded.
33	PMID:32149995	Overall infection was 15/54; infection occurred in 2/29 fractures receiving antibiotics within 24 hours versus 13/17 treated later.	Retrospective; exposure denominators account for 46/54 fractures in the abstract; residual confounding; the timing association is not a safe-delay or causal threshold.	No funding statement indexed in the archived PubMed metadata; not interpreted as unfunded.
34	PMID:31597480	Deep infection predicted operative treatment (OR 34.4); antibiotics were associated with less delayed/nonunion (OR 0.11), and clindamycin had fewer failures than cephalexin (P=.039).	Retrospective treatment-selection confounding, a wide confidence interval, and a setting-specific nonrandom antibiotic comparison.	No funding statement indexed in the archived PubMed metadata; not interpreted as unfunded.
35	DOI:10.4103/atr.atr_9_22	Documents fracture-site nail-bed interposition with nonunion and bipartite nail growth, supporting careful nail-bed assessment and repair.	Single case; no incidence or treatment-effect estimate.	No PubMed funding field; no structured funding statement in the archived bibliographic record; not interpreted as unfunded.
36	PMID:35703237	Mean final angulation was 1.73 degrees, length ratio 97.88%, and ROM ratio 96.92%; no revision-requiring complication occurred, while the crushed group had worse pain, DASH, and ROM.	Small uncontrolled series in which treatment was determined by category/severity, precluding comparative-effectiveness inference.	No funding statement indexed in the archived PubMed metadata; not interpreted as unfunded.
37	PMID:36268384	K-wire fixation appeared associated with less infection, redisplacement, and flexion deformity but more physeal disturbance.	No randomized evidence, mostly indirect or noncomparative studies, confounding by indication, and no meta-analysis.	No funding statement indexed in the archived PubMed metadata; not interpreted as unfunded.
38	PMID:34810026	Debridement within 48 hours was associated with less infection (RR 0.28) and malunion (RR 0.25); antibiotics within 24 hours with less infection (RR 0.21); both interventions with RR 0.30.	Heterogeneous nonrandomized primary studies; timing definitions are analytical rather than safe-delay cutoffs; residual confounding.	No funding statement indexed in the archived PubMed metadata; not interpreted as unfunded.
39	PMID:35311365	Documents a rare intra-articular epiphyseal thumb fracture and its early management/outcome, highlighting the need to exclude an open fracture and restore alignment.	Single case plus narrative review; no frequency or comparative estimate.	No funding statement indexed in the archived PubMed metadata; not interpreted as unfunded.
40	PMID:37791208	A 14-year-old was initially splinted, then received extension-block K-wires because of multiplicity and splint intolerance; at three months, two digits were excellent and one good by Crawford.	Single selected case, adherence-driven treatment change, short follow-up, and no comparator.	No funding statement indexed in the archived PubMed metadata; not interpreted as unfunded.
41	PMID:36607925	Complications were 11.1% versus 23.3% and infections 8.3% versus 20.0%; differences were not statistically significant, with variants trending toward later care and more morbidity.	Single-center, operative-only cohort with selection bias and underpowered nonsignificant comparisons.	No funding statement indexed in the archived PubMed metadata; not interpreted as unfunded.
42	PMID:36974281	Irrigation/debridement, nonabsorbable-suture stabilization, and six weeks of intravenous antibiotics were followed by radiographic healing and infection clearance.	Single case with multiple co-interventions; outcome cannot be attributed to suture fixation.	No funding statement indexed in the archived PubMed metadata; not interpreted as unfunded.

No.	ID	Review-relevant contribution/result	Principal constraint	Funding/support status
43	PMID:37255640	Residual early stiffness progressed to complete DIP autofusion seven years after fixation.	Extremely rare single event; incidence and causality cannot be estimated.	No funding statement indexed in the archived PubMed metadata; not interpreted as unfunded.
44	DOI:10.1016/j.joscr.2023.09.007	They first presented after 8 and 4 days without irrigation or antibiotics and developed osteolysis by days 17 and 11; treatment used debridement, simple external fixation, and antibiotics.	Two cases with multiple interventions; hypothesis-generating only, without a denominator or comparator.	No PubMed funding field; no structured funding statement in the archived bibliographic record; not interpreted as unfunded.
45	PMID:38561523	Union and pain relief occurred in all; 82.1% were excellent/good and 15 fair; no growth or nail deformity was reported; delay and excessive flexion angle correlated with worse outcomes.	Selected surgical cohort with 11 exclusions and no orthosis comparator; retrospective; the abstract's superiority claim is unsupported by the design.	No funding statement indexed in the archived PubMed metadata; not interpreted as unfunded.
46	PMID:39354737	No primary outcome; emphasizes subtle nail-bed injury, infection/growth risk, antibiotics, reduction, and nail-bed repair.	Narrative context without comparative data or reported review methods; full text is non-English unless separately verified.	No funding statement indexed in the archived PubMed metadata; not interpreted as unfunded.
47	PMID:40863716	Closed reduction/splinting failed; CT demonstrated intra-articular displacement; ORIF with K-wire restored congruity with healing and minimal stiffness; the report did not describe a complication during the reported follow-up.	Single complex case; cannot establish the indication for or superiority of ORIF.	No funding statement indexed in the archived PubMed metadata; not interpreted as unfunded.
48	PMID:40687299	Infections were lowest with debridement within eight hours and higher after 24 hours; antibiotics plus debridement were associated with lower infection, and pediatric complication rates were higher.	Heterogeneous nonrandomized aggregate evidence, mixed ages/injuries, and strong preselection/confounding; no causal universal timing threshold.	No funding statement indexed in the archived PubMed metadata; not interpreted as unfunded.
49	PMID:40432862	Eleven scenarios were considered; 17 of 22 resurveyed questions improved to kappa at least 0.70, and consensus included oral antibiotics and hand-surgeon consultation for Seymour fracture.	Level V expert opinion with participant/geographic selection; consensus is not patient-outcome or effectiveness evidence.	No funding statement indexed in the archived PubMed metadata; not interpreted as unfunded.
50	DOI:10.29245/2767-5130/2025/2.1221	No primary outcome; narrative comparison of splinting and surgery, including extension-block K-wire context.	Unclear study selection/appraisal, no quantitative synthesis, and indirect cross-age comparisons; methodologically weak evidence.	No PubMed funding field; no structured funding statement in the archived bibliographic record; not interpreted as unfunded.
51	DOI:10.1016/j.joscr.2024.12.001	Debridement plus vancomycin-impregnated calcium-phosphate paste controlled infection; both growth plates closed prematurely.	Two cases with combined systemic, local, and surgical treatment; the effect of the local carrier cannot be isolated or generalized.	No PubMed funding field; no structured funding statement in the archived bibliographic record; not interpreted as unfunded.
52	PMID:40861033	Describes nail exposure/removal, germinal-matrix release, irrigation/debridement, nail-bed repair, and selective K-wire fixation for instability; no primary outcome series.	Procedural expert guidance rather than comparative evidence; effectiveness and complication claims depend on cited literature.	No funding statement indexed in the archived PubMed metadata; not interpreted as unfunded.
53	DOI:10.47363/JSAR/2025(6)212	No primary outcome; proposes splinting for stable injury and fixation pathways based on morphology and stability.	Search selection/appraisal are insufficiently reported; the algorithm and numerical thresholds are author-derived and unvalidated.	No PubMed funding field; no structured funding statement in the archived bibliographic record; not interpreted as unfunded.
54	PMID:39603863	Both groups had median final lag of 0 degrees and excellent Crawford outcomes, with no significant difference in lag (P=.538) or Crawford result (P=.570).	Small retrospective cohort without a surgical or untreated comparator; selected clinic population.	No funding statement indexed in the archived PubMed metadata; not interpreted as unfunded.
55	PMID:40235410	All received antibiotics within 24 hours; osteomyelitis occurred in one case (3.5%); surgically treated patients had more complications, with no motion loss in the conservative group and restriction in two surgical patients.	Treatment was allocated by reducibility/severity, causing confounding by indication; the abstract reports 18 males plus 11 females despite n=28; no untreated antibiotic comparator.	No funding statement indexed in the archived PubMed metadata; not interpreted as unfunded.

No.	ID	Review-relevant contribution/result	Principal constraint	Funding/support status
56	PMID:41289484	No primary outcome; summarizes immobilization and proposed operative indications.	Expert/educational synthesis; thresholds have not been prospectively validated as universal pediatric rules.	No funding statement indexed in the archived PubMed metadata; not interpreted as unfunded.
57	PMID:41065608	No primary outcome; update on classification, surgical indications, and chronic-treatment context.	Narrative source selection without new comparative data.	No funding statement indexed in the archived PubMed metadata; not interpreted as unfunded.
58	PMID:42434507	No primary outcome; supports nail-unit signs, dedicated lateral imaging, open-fracture principles, and selective stabilization for persistent instability.	Recommendations derive largely from retrospective series and cases; no validated diagnostic rule or fixation/antibiotic comparison.	No funding statement indexed in the archived PubMed metadata; not interpreted as unfunded.
59	PMID:42058391	No primary outcome; supports finger radiographs, functional examination, immediate-management principles, and hand-specialist follow-up.	Broad review plus expert opinion rather than a Seymour-specific comparative study.	No funding statement indexed in the archived PubMed metadata; not interpreted as unfunded.
60	DOI:10.4103/tjps.tjps _85_25	Radiographic angle improved from 21.4° to 10.4° ($P<0.05$); extension lag was 0° and Crawford outcome was excellent in all 20. No major complication, recurrence, pin-tract irritation, or temporary stiffness was observed in this selected series.	Small selected surgical cohort without a comparator; mixed bony/tendinous injuries and categorical ceiling effects. The absence of an observed event establishes neither safety nor surgical superiority.	No PubMed funding field; no structured funding statement in the archived bibliographic record; not interpreted as unfunded.

Funding summary: one of 60 sources carried an extractable funding acknowledgement (PMID 24209954: NIAMS/NIH support including 2R01 AR047328-06 and K24 AR053120). Fifty-two other PMID-linked reports had no funding statement in the archived PubMed metadata; seven DOI-only reports had no PubMed funding field or structured funding statement in the archived bibliographic record. These categories mean not indexed/not extractable, not unfunded.

S2.2. Design-specific limitations (not a formal critical appraisal)

Formal source-level critical appraisal was not undertaken and no source was excluded on quality grounds. The source-level principal-constraint column above is a narrative evidence-context map, not a scored or validated risk-of-bias result.

Evidence type	Descriptive context	Use	Main limitation
Systematic reviews	Review design and source base described	Kiely directly addresses Seymour management; El Sewify addresses broader open distal-phalanx surgery.	Dependent on heterogeneous non-randomized primary evidence.
Cohorts/comparative series	Design, sample, allocation, and follow-up described when available	Best information on timing, infection, treatment completeness, stability, and pediatric mallet outcomes.	Mostly retrospective; confounding by indication, selection, and incomplete follow-up.
Case series/reports	Distinct phenotype or complication mapped	Retained when anatomy, mechanism, or complication was not otherwise represented.	Cannot estimate frequency or comparative effectiveness.
Narrative/clinical reviews	No-primary-data status stated	Provide terminology and clinical context.	Conclusions depend on non-systematic source selection and interpretation.
External standards	Clinical concordance only	BSSH and RCH support open-injury urgency and closed-mallet splint principles.	Not counted among the 60 included research sources; not a substitute for comparative evidence.

The evidence base was methodologically limited; no formal certainty grading was performed. Timing and antibiotic signals are directionally consistent but non-causal; no diagnostic-accuracy study was found; no universal pediatric fragment-size, displacement, or instability threshold was validated.

S2.3. Reconciled database candidate ledger (161 unique records)

This ledger was reconstructed deterministically from the immutable PubMed and OpenAlex exports after DOI/PMID/title deduplication. It reports the final reconciled consensus outcome. The 84 title/abstract exclusions are the set difference after identifying the 77 reports assessed; individual title/abstract reasons, reviewer-level votes, timestamps, and disagreement counts were not retained and were not reconstructed.

No.	Source hit(s)	Canonical ID	Year	Title
1	PubMed A; OpenAlex Seymour full-text	PMID:42434507	2026	Seymour Fractures Revisited: Recognition and Management of an Open Physeal Distal Phalanx Injury.
2	PubMed A	PMID:42058391	2026	Management of Pediatric Hand Injuries in the Acute Setting Part 2: Fractures and Dislocations.
3	PubMed A; OpenAlex Seymour full-text	PMID:41289484	2026	Physeal Phalanx Fractures: To Fix or Not To Fix?
4	PubMed A; OpenAlex Seymour full-text	PMID:41106872	2025	Minor Trauma that Ends up Being a Major Headache.
5	PubMed A	PMID:41065608	2026	Pediatric Finger Fractures: Preventing Big Problems After Small Fractures.
6	PubMed A; PubMed B; OpenAlex Seymour full-text	PMID:40861033	2025	Management of Seymour (Distal Phalangeal) Fractures with Nail Bed Involvement.
7	PubMed A; OpenAlex Seymour full-text	PMID:40838653	2025	Seymour fracture.
8	PubMed A	PMID:40687299	2025	A systematic review of surgical management for open fractures of the distal phalanx: What is the evidence?
9	PubMed A	PMID:40501358	2025	Radiographic characteristics of epiphyseal ossification centres of the thumb in patients with radial polydactyly.
10	PubMed A; OpenAlex Seymour full-text	PMID:40432862	2025	It's Just a Fingertip! Yet Controversy Exists: Standardizing a Treatment Pathway for Pediatric Fingertip Injuries.

No.	Source hit(s)	Canonical ID	Year	Title
11	PubMed A; OpenAlex Seymour full-text	PMID:40235410	2025	The silent threat under the nail: Evaluation of Seymour fractures in pediatric patients.
12	PubMed A; OpenAlex Seymour full-text	PMID:40040758	2025	Seymour fracture management and functional outcome assessment: a case report.
13	PubMed A	PMID:39435769	2025	Accelerated Frostbite-induced Acroosteolysis in Pediatric Insensate Hand: A Case Report and Literature Review.
14	PubMed A; OpenAlex Seymour full-text	PMID:39354737	2024	[The Seymour fracture - a fracture that easily can be missed].
15	PubMed A	PMID:38589862	2024	Clinical analysis of 1301 children with hand and foot fractures and growth plate injuries.
16	PubMed A; OpenAlex Seymour full-text	PMID:37704235	2023	Approach to nail trauma for primary care physicians.
17	PubMed A; OpenAlex Seymour full-text	PMID:36974281	2023	Suture Fixation of Subacute Pediatric Seymour Fractures.
18	PubMed A; OpenAlex Seymour full-text	PMID:36607925	2023	Complication Rates in Patients With Classic and Radiographic Variants of Seymour Fractures.
19	PubMed A; OpenAlex Seymour full-text	PMID:36268384	2022	Does K-wire fixation improve outcomes in children with a Seymour fracture?
20	PubMed A; OpenAlex Seymour full-text	PMID:35703237	2022	Treatment Strategies for Acute Seymour Fractures in Children and Adolescents: Including Crushed Open Fractures.

No.	Source hit(s)	Canonical ID	Year	Title
21	PubMed A; OpenAlex Seymour full-text	PMID:35489831	2022	Reprint of: seymour fracture: A particularly tuft diagnosis.
22	PubMed A; OpenAlex Seymour full-text	PMID:35311365	2022	Salter-Harris Type III Fracture of the Distal Phalanx: A Rare Juxtaphyseal Variant.
23	PubMed A; OpenAlex Seymour full-text	PMID:34842086	2021	Seymour fracture: Better do not underestimate it.
24	PubMed A; OpenAlex Seymour full-text	PMID:34810026	2022	Management of Seymour Fractures in Children and Adolescents: A Systematic Review and Meta-Analysis.
25	PubMed A; PubMed B; OpenAlex Seymour full-text	PMID:34557438	2021	Seymour's Fracture in a 13-years-old Child: A Case Report.
26	PubMed A; OpenAlex Seymour full-text	PMID:37020613	2023	Phalangeal and Metacarpal Fractures in Children: A 10-Year Comparison of Factors Affecting Functional Outcomes in 313 Patients.
27	PubMed A; OpenAlex Seymour full-text	PMID:34104672	2021	A rare case of Seymour fracture in an adult with non-fused growth plates.
28	PubMed A; OpenAlex Seymour full-text	PMID:33508522	2021	Acute Seymour fractures in children/adolescents overlooked as a simple nail injury: Reduction without pinning.
29	PubMed A; PubMed B; OpenAlex Seymour full-text	PMID:33133852	2020	Seymour Fracture in a Pediatric Patient: A Case Report.
30	PubMed A; OpenAlex Seymour full-text	PMID:32586465	2020	Pediatric Hand Infections.

No.	Source hit(s)	Canonical ID	Year	Title
31	PubMed A; OpenAlex Seymour full-text	PMID:32576259	2020	The optimal management of Seymour fractures in children and adolescents: a systematic review protocol.
32	PubMed A; OpenAlex Seymour full-text	PMID:32149995	2021	Seymour Fractures: A Retrospective Review of Infection Rates, Treatment and Timing of Antibiotic Administration.
33	PubMed A; OpenAlex Seymour full-text	PMID:31690204	2019	Open Infected Seymour Fracture.
34	PubMed A; OpenAlex Seymour full-text	PMID:31597480	2021	Delayed Presentation of Seymour Fractures: A Single Institution Experience and Management Recommendations.
35	PubMed A; OpenAlex Seymour full-text	PMID:31103087	2019	Pediatric Hand and Wrist Fractures.
36	PubMed A; PubMed B; OpenAlex Seymour full-text	PMID:30759479	2019	The patterns of phalangeal fractures in children and adolescents: a review of 512 cases.
37	PubMed A; OpenAlex Seymour full-text	PMID:30358692	2019	Treatment of Acute Seymour Fractures.
38	PubMed A	PMID:29667031	2018	The prevalence and ossification pattern of the biphalangeal and triphalangeal lateral toes.
39	PubMed A; PubMed B	PMID:29399000	2018	Outcomes Following Operative Treatment of Adolescent Mallet Fractures.
40	PubMed A; OpenAlex Seymour full-text	PMID:28735119	2017	A case series of pediatric seymour fractures related to hoverboards: Increasing trend with changing lifestyle.

No.	Source hit(s)	Canonical ID	Year	Title
41	PubMed A; OpenAlex Seymour full-text	PMID:28110548	2018	Musculoskeletal Injuries Resulting from Use of Hoverboards: Safety Concerns With an Unregulated Consumer Product.
42	PubMed A	PMID:28058484	2017	Hoverboards: spectrum of injury and association with an uncommon fracture.
43	PubMed A; PubMed B; OpenAlex Seymour full-text	PMID:27490217	2016	Problematic Pediatric Hand and Wrist Fractures.
44	PubMed A; OpenAlex Seymour full-text	PMID:29786288	2016	[SURGICAL TREATMENT OF Seymour FRACTURES IN CHILDREN AND ADOLESCENTS].
45	PubMed A	PMID:26895519	2016	[Epiphyseal Bar Resection for Correction of Clinodactyly].
46	PubMed A; OpenAlex Seymour full-text	PMID:26327401	2017	The High Risk of Infection With Delayed Treatment of Open Seymour Fractures: Salter-Harris I/II or Juxta-epiphyseal Fractures of the Distal Phalanx With Associated Nailbed Laceration.
47	PubMed A; PubMed B	PMID:26279261	2015	Bony mallet finger without epiphyseal plate injury in childhood.
48	PubMed A	PMID:24841396	2014	Images in emergency medicine. A jammed finger and bloody nail. Seymour fracture.
49	PubMed A; OpenAlex Seymour full-text	PMID:24206995	2013	Seymour fractures.
50	PubMed A	PMID:24201073	2014	Is progressive lengthening of the distal phalanx of a child's index finger possible and safe?

No.	Source hit(s)	Canonical ID	Year	Title
51	PubMed A; PubMed B	PMID:24194615	2014	Distal phalanx fracture in adults: Seymour-type fracture.
52	PubMed A; OpenAlex Seymour full-text	PMID:23351909	2013	Seymour fractures: retrospective analysis and therapeutic considerations.
53	PubMed A; PubMed B	PMID:15958901	2005	Base of distal phalanx fracture in children: a mallet finger mimic.
54	PubMed A	PMID:9763283	1998	Physeal growth arrest of the distal phalanx of the thumb in an adolescent pianist: a case report.
55	PubMed A	PMID:9620197	1998	Physeal growth arrest of the distal phalanx of the thumb in an adolescent pianist: a case report.
56	PubMed A	PMID:1572921	1992	Reconstruction of traumatic absence of the thumb by microvascular free tissue transfer from the foot.
57	PubMed A	PMID:3408289	1988	[Infection of the tendon sheath following an open epiphyseal fracture. Apropos of a case].
58	PubMed A; PubMed B	PMID:7364328	1980	Displaced epiphyseal plate of the terminal phalanx in a child.
59	PubMed A	PMID:488788	1979	Fractures of the phalanges of the hand in children.
60	PubMed B	PMID:41253575	2025	What is known from the existing literature about the treatment of mallet injury using 3D-printed splints? A scoping review.

No.	Source hit(s)	Canonical ID	Year	Title
61	PubMed B; OpenAlex pediatric mallet title	PMID:40863716	2025	Open Reduction and Internal Fixation of a Volar Displaced Salter-Harris III Mallet Fracture in a Pediatric Patient: A Case Report.
62	PubMed B	PMID:40760545	2025	Ishiguro technique percutaneous metallic bone needle closed reduction for Judet IV radial head fractures in children: A case report (CARE-compliant).
63	PubMed B	PMID:29494114	2026	Subungual Hematoma Drainage
64	PubMed B; OpenAlex pediatric mallet title	PMID:40073800	2025	Finger osteochondroma presenting as non-traumatic mallet finger in pediatric: A case report.
65	PubMed B	PMID:39603863	2025	The outcome difference between acute bony and tendinous mallet fingers treated conservatively in children.
66	PubMed B	PMID:39311252	2024	Reconstruction of Chronic Soft Tissue Mallet Fingers: Outcomes of Step-Plasty vs. Purse-String Suture.
67	PubMed B	PMID:38561523	2024	Retrospective study of Ishiguro's technique for mallet bone finger in children: long-term follow-up and analysis of predictors in outcomes.
68	PubMed B	PMID:37791208	2023	Multiple Bony Mallet Finger Injuries in One Hand of a 14-Year-Old Boy.
69	PubMed B	PMID:37255640	2023	Mallet Finger Autofusion in Adolescent Post-Pin Fixation Surgery - A Case Report.
70	PubMed B	PMID:35377856	2022	[Case reports of pediatric electrical finger burn injuries' management and late- onset complications].

No.	Source hit(s)	Canonical ID	Year	Title
71	PubMed B	PMID:30808392	2019	Scar overlapping suture for treating chronic tendinous mallet finger in children.
72	PubMed B; OpenAlex pediatric mallet title	PMID:30730419	2019	Comparison of Percutaneous Reduction and Pin Fixation in Acute and Chronic Pediatric Mallet Fractures.
73	PubMed B	PMID:30730385	2021	An Evidence-Based Approach to Casting and Orthosis Management of the Pediatric, Adolescent, and Young Adult Population for Injuries of the Upper Extremity: A Review Article.
74	PubMed B; OpenAlex pediatric mallet title	PMID:29776724	2018	Outcomes of Splinting in Pediatric Mallet Finger.
75	PubMed B	PMID:28968312	2017	Mallet Finger in a Toddler: A Rare But Easily Missed Injury.
76	PubMed B	PMID:28719993	2018	Comparison of Treatment Results for Mallet Finger Fractures in Children Between Low-Intensity Pulsed Ultrasound Stimulation and Ishiguro's Method.
77	PubMed B	PMID:28465015	2017	Complications of Distal Phalanx Fractures in Children.
78	PubMed B	PMID:28196434	2017	The anatomy of Wassel type IV-D thumb duplication.
79	PubMed B	PMID:18548355	2008	[The mallet finger in children and adolescents].
80	PubMed B	PMID:18157050	2008	The results of tenodermodesis for severe chronic mallet finger deformity in children.

No.	Source hit(s)	Canonical ID	Year	Title
81	PubMed B	PMID:18090832	2007	Tenodermodesis for chronic mallet finger deformities in children.
82	PubMed B	PMID:11418917	2001	A congenital hand deformity: Dupuytren's disease.
83	PubMed B	PMID:11077860	2000	[Mini-invasive osteosynthesis in injuries of the finger phalangeal bones in children].
84	PubMed B	PMID:1573000	1992	Treatment of chronic mallet finger deformity in children by tenodermodesis.
85	OpenAlex Seymour full-text	DOI:10.1097/bpo.0000000000001676	2020	Pediatric Seymour Fractures of the Toe
86	OpenAlex Seymour full-text	DOI:10.1097/bpo.0000000000001632	2020	Suture Stabilization of Open Physeal Fractures of the Great Toe Distal Phalanx (Seymour Fracture)
87	OpenAlex Seymour full-text	DOI:10.1007/978-3-319-29980-8_60	2020	Seymour Fracture (Open Physeal Fracture of the Distal Phalanx)
88	OpenAlex Seymour full-text	DOI:10.1016/j.ajem.2017.02.015	2017	Open physeal fracture of the distal phalanx of the hallux
89	OpenAlex Seymour full-text	DOI:10.1097/bto.0b013e3181b58eb2	2009	Pediatric Hand Fractures
90	OpenAlex Seymour full-text	DOI:10.1136/bcr-2021-244985	2021	Pinckney fracture: do not underestimate trauma of the distal phalanx of the hallux

No.	Source hit(s)	Canonical ID	Year	Title
91	OpenAlex Seymour full-text	DOI:10.1007/978-3-319-29980-8_57	2020	Nailbed Injuries
92	OpenAlex Seymour full-text	DOI:10.1016/j.nurpra.2020.06.017	2020	Diagnosis and Management of Common Pediatric Fingertip Trauma
93	OpenAlex Seymour full-text	DOI:10.29315/gm.v5i2.165	2018	Infected Seymour-Type Fracture
94	OpenAlex Seymour full-text	DOI:10.1136/bmj.328.7444.900-c	2004	William Sim Munro
95	OpenAlex Seymour full-text	DOI:10.1007/978-3-319-56651-1_10	2018	The Jammed Finger
96	OpenAlex Seymour full-text	DOI:10.1007/978-1-4614-8515-5_44	2014	Pediatric Phalanx Fractures: Evaluation and Management
97	OpenAlex Seymour full-text	DOI:10.1007/978-1-4614-8758-6_44-1	2014	Pediatric Phalanx Fractures: Evaluation and Management
98	OpenAlex Seymour full-text	DOI:10.1016/j.visj.2021.100980	2021	Seymour fracture: A particularly tuft diagnosis
99	OpenAlex Seymour full-text	https://openalex.org/W3019939436	2011	Seymour Fracture Of DIstal Phalanx In A ChIld
100	OpenAlex Seymour full-text	DOI:10.1093/bjs/znab259.946	2021	1070 A Systematic Review of Seymour Fracture Management

No.	Source hit(s)	Canonical ID	Year	Title
101	OpenAlex Seymour full-text	DOI:10.2106/jbjs.st.24.00024	2025	Surgical Treatment of Pediatric Seymour Fractures of the Great Toe
102	OpenAlex Seymour full-text; OpenAlex pediatric mallet title	DOI:10.1007/978-3-031-32072-9_6	2023	Seymour Fractures and Bony Mallet Fractures in the Pediatric Population
103	OpenAlex Seymour full-text	DOI:10.1007/978-3-319-28226-8_60-1	2017	Seymour Fracture (Open Physeal Fracture of the Distal Phalanx)
104	OpenAlex Seymour full-text	DOI:10.1016/j.jpeds.2021.01.056	2021	Physical Examination Identifies Seymour Fractures of the Great Toe
105	OpenAlex Seymour full-text	DOI:10.1016/j.jhsa.2014.06.109	2014	Is There a Higher Risk of Infection With Delayed Treatment of Pediatric Seymour Fractures?
106	OpenAlex Seymour full-text	DOI:10.21203/rs.3.rs-21070/v2	2020	The optimal management of Seymour fractures in children: a systematic review protocol
107	OpenAlex Seymour full-text	DOI:10.1097/bpo.0000000000002820	2024	A Neglected Fracture: Treatment and Infection of Seymour Fractures of the Hallux in the Pediatric Population
108	OpenAlex Seymour full-text	DOI:10.1016/j.joscr.2023.09.007	2023	Acute osteomyelitis after the physeal injury of the distal phalanx of the hand (Seymour fracture): A report of two cases
109	OpenAlex Seymour full-text	DOI:10.21203/rs.3.rs-21070/v1	2020	Protocol for a systematic review of the incidence of infection in operative vs non-operative management of Seymour fractures in children and adolescents
110	OpenAlex Seymour full-text	DOI:10.1016/j.joscr.2024.12.001	2025	Two cases of treatment of the seymour fracture complicated by pyogenic arthritis with antibiotic-impregnated calcium phosphate paste granules

No.	Source hit(s)	Canonical ID	Year	Title
111	OpenAlex Seymour full-text	DOI:10.1017/cem.2020.293	2020	P087: A comparison of how emergency physicians and plastic surgeons evaluate and triage pediatric hand fractures: a prospective trial of the Calgary Kids' Hand Rule
112	OpenAlex Seymour full-text	DOI:10.4103/atr.atr_9_22	2022	Bipartite nail deformity due to seymour's fracture in a pediatric patient
113	OpenAlex Seymour full-text	DOI:10.1016/j.jhsa.2013.09.011	2013	Journal CME Questions
114	OpenAlex Seymour full-text	DOI:10.1055/b-0040-177446	2020	31 Distal Phalanx Fractures: Percutaneous Pinning and Open Reduction Internal Fixation
115	OpenAlex Seymour full-text	DOI:10.1007/978-981-10-4400-7_22	2019	Miscellaneous
116	OpenAlex Seymour full-text	DOI:10.1007/978-3-319-28226-8_57-1	2019	Nailbed Injuries
117	OpenAlex Seymour full-text	DOI:10.1542/pir.2015-0160	2017	Acute and Chronic Growth Plate Injuries
118	OpenAlex Seymour full-text	DOI:10.1302/2048-0105.121.3600028	2023	Wrist & Hand
119	OpenAlex Seymour full-text	DOI:10.1007/s12178-017-9395-6	2017	A review of mallet finger and jersey finger injuries in the athlete
120	OpenAlex Seymour full-text	DOI:10.1542/pir.36.5.184	2015	The Limping Child

No.	Source hit(s)	Canonical ID	Year	Title
121	OpenAlex Seymour full-text	DOI:10.1542/pir.2021-004987	2022	You Can Handle It: Pediatric Hand Injuries from Toddlers to Teens
122	OpenAlex Seymour full-text	DOI:10.1007/s12178-012-9146-7	2012	Pediatric hand and wrist injuries
123	OpenAlex Seymour full-text	DOI:10.62547/rurz7083	2023	Decreasing Trend in Upper Extremity Surfing Injuries Presenting to United States Emergency Departments – A 20-Year Analysis
124	OpenAlex Seymour full-text	DOI:10.3390/children10091446	2023	Traffic Accidents in Children and Adolescents: A Complex Orthopedic and Medico-Legal Approach
125	OpenAlex Seymour full-text	DOI:10.5772/intechopen.76658	2018	Crush Injuries of the Hand Part I: History, Mechanism and Pathomechanics
126	OpenAlex Seymour full-text	DOI:10.1055/s-0037-1602414	2017	Emergency and Trauma of Hand and Wrist
127	OpenAlex Seymour full-text	DOI:10.1007/s00264-023-05858-y	2023	News in paediatric orthopaedic surgery: an overview of the latest advances in paediatric orthopaedics and traumatology (2020–2023)
128	OpenAlex Seymour full-text	DOI:10.25214/28056272.1180	2021	Fractura osteocondral abierta del penacho de la falange distal en punta de dedo en niños: una lesión frecuentemente no diagnosticada
129	OpenAlex Seymour full-text	DOI:10.52312/jdrs.2023.994	2023	The effects of cephalexin on fracture healing in a rat femur fracture model
130	OpenAlex Seymour full-text	DOI:10.7759/cureus.17246	2021	Development of an Effective Treatment Algorithm for the Stubbed Great Toe

No.	Source hit(s)	Canonical ID	Year	Title
131	OpenAlex Seymour full-text	DOI:10.1007/978-3-319-29980-8	2020	Pediatric Orthopedic Trauma Case Atlas
132	OpenAlex Seymour full-text	DOI:10.3389/fped.2023.1118954	2023	Assessing the risks of children with preoperative comorbidities undergoing comminuted fracture surgery
133	OpenAlex Seymour full-text	DOI:10.46747/cfp.720293	2026	Approach to mallet finger injury
134	OpenAlex Seymour full-text	DOI:10.52965/001c.125898	2024	Comminuted, displaced, and angulated distal radial and ulnar metaphyseal fractures after a trampoline park accident
135	OpenAlex Seymour full-text	DOI:10.13112/pc.442	2019	Problematični prijelomi članaka prstiju šake u dječjoj dobi
136	OpenAlex Seymour full-text	DOI:10.1007/978-3-031-32072-9	2023	Pediatric and Adult Hand Fractures
137	OpenAlex Seymour full-text	DOI:10.52581/1814-1471/93/10	2025	Eponyms in hand injury surgery
138	OpenAlex Seymour full-text	DOI:10.46747/cfp.6909615	2023	Approche à l’égard des traumatismes unguéaux pour les médecins de soins primaires
139	OpenAlex Seymour full-text	DOI:10.1016/j.jposna.2026.100365	2026	Management of Pediatric Hand Injuries in the Acute Setting Part 3: Lacerations and Fingertip Injuries
140	OpenAlex Seymour full-text	DOI:10.18019/1028-4427-2021-27-3-322-324	2021	Technical points of Ilizarov external mini-fixator for the treatment of phalanx fractures

No.	Source hit(s)	Canonical ID	Year	Title
141	OpenAlex Seymour full-text	DOI:10.7759/cureus.87790	2025	Knee Pain: The Fine Line Between Malignant and Benign
142	OpenAlex Seymour full-text	DOI:10.18019/1028-4427-2021-27-3-319-321	2021	Outcomes using the Ilizarov external mini-fixator for Monteggia fractures in children
143	OpenAlex Seymour full-text	DOI:10.52965/001c.125161	2024	Distal Phalanx Fracture Secondary to Nail Gun Injury
144	OpenAlex Seymour full-text	DOI:10.52783/jes.8388	2024	Design and Development of Roadside Wheeler
145	OpenAlex Seymour full-text	DOI:10.7759/cureus.104513	2026	Outcome of Open Distal Tibial Physeal Fractures: A Descriptive Report of Two Cases
146	OpenAlex Seymour full-text	DOI:10.1016/j.jhsa.2026.04.010	2026	Good Enough: When to Avoid Perfection and Accept Adequate Surgery Results in Pediatric Hand Surgery
147	OpenAlex Seymour full-text	DOI:10.52312/jdrs.2026.2698	2026	Juxta-epiphyseal fractures at the base of the proximal phalanx of the finger in pediatric patients
148	OpenAlex Seymour full-text	DOI:10.14292/totbid.dergisi.2019.51	2019	Karpal kemikler ve el (metakarp ve falanks) kırıkları ve çıkıkları
149	OpenAlex Seymour full-text	DOI:10.46747/cfp.7202e30	2026	Approche face à un doigt en maillet
150	OpenAlex Seymour full-text	DOI:10.37811/cl_rcm.v9i2.17176	2025	Fracturas en el Cartílago de Crecimiento: Desafíos Diagnósticos y Tratamientos en la Niñez

No.	Source hit(s)	Canonical ID	Year	Title
151	OpenAlex Seymour full-text	DOI:10.13107/jocr.2023.v13.i11.4014	2023	Closed Mallet Thumb Injury-A Rare Case Report
152	OpenAlex Seymour full-text	DOI:10.1007/978-3-032-17324-9	2026	Fracture Finder: A Practical Guide to Interpreting Upper and Lower Limb X-Rays for Radiographers
153	OpenAlex Seymour full-text	DOI:10.14292/totbid.dergisi.2014.13	2014	Distal falanks kırıkları
154	OpenAlex Seymour full-text	DOI:10.5578/totbid.dergisi.2024.36	2024	Çocuklarda ön kol, el bileği ve el kırıkları
155	OpenAlex Seymour full-text	DOI:10.35631/ijepc.1163033	2026	AI-DRIVEN REFORM OF CAREER PLANNING IN CHINESE HIGHER EDUCATION: A SYSTEMATIC LITERATURE REVIEW
156	OpenAlex pediatric mallet title	DOI:10.4103/tjps.tjps_85_25	2026	Clinical and Radiological Outcomes of Surgically Treated Pediatric Mallet Finger
157	OpenAlex pediatric mallet title	DOI:10.1542/peds.141.1_meetingabstract.665	2018	Comparison of Percutaneous Reduction and Pin Fixation in Chronic and Acute Pediatric Mallet Fractures
158	OpenAlex pediatric mallet title	DOI:10.1542/peds.141.1ma7.665	2018	Comparison of Percutaneous Reduction and Pin Fixation in Chronic and Acute Pediatric Mallet Fractures
159	OpenAlex pediatric mallet title	DOI:10.29245/2767-5130/2025/2.1221	2025	Bony Mallet Finger: A Comparative Review of Approaches in Pediatric vs. Adult Populations
160	OpenAlex pediatric mallet title	DOI:10.47363/jsar/2025(6)212	2025	Treatment of Pediatric Bone Mallet Finger: The Current State of the Art and a Proposal of Algorithm Treatment

No.	Source hit(s)	Canonical ID	Year	Title
161	OpenAlex pediatric mallet title	DOI:10.7197/cmj.v37i2.5000079536	2015	Evaluation of clinical outcome of acute mallet finger deformity managed conservatively in pediatric patients

S2.3 continued. Reconciled database decisions

No.	Canonical ID	Stage	Final consensus decision	Reason
1	PMID:42434507	Report assessed	Included	Met the prespecified eligibility criteria and was charted in the evidence map.
2	PMID:42058391	Report assessed	Included	Met the prespecified eligibility criteria and was charted in the evidence map.
3	PMID:41289484	Report assessed	Included	Met the prespecified eligibility criteria and was charted in the evidence map.
4	PMID:41106872	Report assessed	Excluded after report assessment	Multi-trauma review without extractable target synthesis
5	PMID:41065608	Report assessed	Included	Met the prespecified eligibility criteria and was charted in the evidence map.
6	PMID:40861033	Report assessed	Included	Met the prespecified eligibility criteria and was charted in the evidence map.
7	PMID:40838653	Report assessed	Excluded after report assessment	Clinical image without extractable data
8	PMID:40687299	Report assessed	Included	Met the prespecified eligibility criteria and was charted in the evidence map.
9	PMID:40501358	Title/abstract	Excluded at title/abstract	Final consensus exclusion at title/abstract stage; a more granular record-level reason was not retained.
10	PMID:40432862	Report assessed	Included	Met the prespecified eligibility criteria and was charted in the evidence map.

No.	Canonical ID	Stage	Final consensus decision	Reason
11	PMID:40235410	Report assessed	Included	Met the prespecified eligibility criteria and was charted in the evidence map.
12	PMID:40040758	Report assessed	Excluded after report assessment	Routine single case without a distinct phenotype/complication
13	PMID:39435769	Title/abstract	Excluded at title/abstract	Final consensus exclusion at title/abstract stage; a more granular record-level reason was not retained.
14	PMID:39354737	Report assessed	Included	Met the prespecified eligibility criteria and was charted in the evidence map.
15	PMID:38589862	Title/abstract	Excluded at title/abstract	Final consensus exclusion at title/abstract stage; a more granular record-level reason was not retained.
16	PMID:37704235	Report assessed	Excluded after report assessment	General nail-trauma review without target result
17	PMID:36974281	Report assessed	Included	Met the prespecified eligibility criteria and was charted in the evidence map.
18	PMID:36607925	Report assessed	Included	Met the prespecified eligibility criteria and was charted in the evidence map.
19	PMID:36268384	Report assessed	Included	Met the prespecified eligibility criteria and was charted in the evidence map.
20	PMID:35703237	Report assessed	Included	Met the prespecified eligibility criteria and was charted in the evidence map.

No.	Canonical ID	Stage	Final consensus decision	Reason
21	PMID:35489831	Report assessed	Excluded after report assessment	Reprint/duplicate publication
22	PMID:35311365	Report assessed	Included	Met the prespecified eligibility criteria and was charted in the evidence map.
23	PMID:34842086	Report assessed	Included	Met the prespecified eligibility criteria and was charted in the evidence map.
24	PMID:34810026	Report assessed	Included	Met the prespecified eligibility criteria and was charted in the evidence map.
25	PMID:34557438	Report assessed	Excluded after report assessment	Routine single case without a distinct phenotype/complication
26	PMID:37020613	Report assessed	Excluded after report assessment	Broad hand cohort without separable target result
27	PMID:34104672	Report assessed	Excluded after report assessment	Adult population
28	PMID:33508522	Report assessed	Included	Met the prespecified eligibility criteria and was charted in the evidence map.
29	PMID:33133852	Report assessed	Excluded after report assessment	Routine single case without a distinct phenotype/complication
30	PMID:32586465	Report assessed	Excluded after report assessment	General hand-infection review without target cohort

No.	Canonical ID	Stage	Final consensus decision	Reason
31	PMID:32576259	Report assessed	Excluded after report assessment	Protocol without results
32	PMID:32149995	Report assessed	Included	Met the prespecified eligibility criteria and was charted in the evidence map.
33	PMID:31690204	Report assessed	Excluded after report assessment	Adult case (24 years)
34	PMID:31597480	Report assessed	Included	Met the prespecified eligibility criteria and was charted in the evidence map.
35	PMID:31103087	Report assessed	Excluded after report assessment	General hand/wrist-fracture review without separable target synthesis
36	PMID:30759479	Report assessed	Excluded after report assessment	Broad phalangeal cohort without separable target result
37	PMID:30358692	Report assessed	Included	Met the prespecified eligibility criteria and was charted in the evidence map.
38	PMID:29667031	Title/abstract	Excluded at title/abstract	Final consensus exclusion at title/abstract stage; a more granular record-level reason was not retained.
39	PMID:29399000	Report assessed	Included	Met the prespecified eligibility criteria and was charted in the evidence map.
40	PMID:28735119	Report assessed	Included	Met the prespecified eligibility criteria and was charted in the evidence map.

No.	Canonical ID	Stage	Final consensus decision	Reason
41	PMID:28110548	Title/abstract	Excluded at title/abstract	Final consensus exclusion at title/abstract stage; a more granular record-level reason was not retained.
42	PMID:28058484	Title/abstract	Excluded at title/abstract	Final consensus exclusion at title/abstract stage; a more granular record-level reason was not retained.
43	PMID:27490217	Report assessed	Included	Met the prespecified eligibility criteria and was charted in the evidence map.
44	PMID:29786288	Report assessed	Included	Met the prespecified eligibility criteria and was charted in the evidence map.
45	PMID:26895519	Title/abstract	Excluded at title/abstract	Final consensus exclusion at title/abstract stage; a more granular record-level reason was not retained.
46	PMID:26327401	Report assessed	Included	Met the prespecified eligibility criteria and was charted in the evidence map.
47	PMID:26279261	Report assessed	Included	Met the prespecified eligibility criteria and was charted in the evidence map.
48	PMID:24841396	Report assessed	Excluded after report assessment	Clinical image without extractable data
49	PMID:24206995	Report assessed	Included	Met the prespecified eligibility criteria and was charted in the evidence map.
50	PMID:24201073	Title/abstract	Excluded at title/abstract	Final consensus exclusion at title/abstract stage; a more granular record-level reason was not retained.

No.	Canonical ID	Stage	Final consensus decision	Reason
51	PMID:24194615	Report assessed	Excluded after report assessment	Adult population
52	PMID:23351909	Report assessed	Included	Met the prespecified eligibility criteria and was charted in the evidence map.
53	PMID:15958901	Report assessed	Included	Met the prespecified eligibility criteria and was charted in the evidence map.
54	PMID:9763283	Report assessed	Excluded after report assessment	Bibliographic duplicate of PMID 9620197
55	PMID:9620197	Report assessed	Excluded after report assessment	Repetitive overuse rather than target acute trauma
56	PMID:1572921	Title/abstract	Excluded at title/abstract	Final consensus exclusion at title/abstract stage; a more granular record-level reason was not retained.
57	PMID:3408289	Report assessed	Included	Met the prespecified eligibility criteria and was charted in the evidence map.
58	PMID:7364328	Report assessed	Included	Met the prespecified eligibility criteria and was charted in the evidence map.
59	PMID:488788	Report assessed	Included	Met the prespecified eligibility criteria and was charted in the evidence map.
60	PMID:41253575	Report assessed	Excluded after report assessment	Mixed 3D-splint review without separable pediatric target result

No.	Canonical ID	Stage	Final consensus decision	Reason
61	PMID:40863716	Report assessed	Included	Met the prespecified eligibility criteria and was charted in the evidence map.
62	PMID:40760545	Title/abstract	Excluded at title/abstract	Final consensus exclusion at title/abstract stage; a more granular record-level reason was not retained.
63	PMID:29494114	Title/abstract	Excluded at title/abstract	Final consensus exclusion at title/abstract stage; a more granular record-level reason was not retained.
64	PMID:40073800	Report assessed	Excluded after report assessment	Non-traumatic osteochondroma
65	PMID:39603863	Report assessed	Included	Met the prespecified eligibility criteria and was charted in the evidence map.
66	PMID:39311252	Report assessed	Excluded after report assessment	Chronic reconstruction; population outside target
67	PMID:38561523	Report assessed	Included	Met the prespecified eligibility criteria and was charted in the evidence map.
68	PMID:37791208	Report assessed	Included	Met the prespecified eligibility criteria and was charted in the evidence map.
69	PMID:37255640	Report assessed	Included	Met the prespecified eligibility criteria and was charted in the evidence map.
70	PMID:35377856	Title/abstract	Excluded at title/abstract	Final consensus exclusion at title/abstract stage; a more granular record-level reason was not retained.

No.	Canonical ID	Stage	Final consensus decision	Reason
71	PMID:30808392	Report assessed	Excluded after report assessment	Chronic reconstruction
72	PMID:30730419	Report assessed	Included	Met the prespecified eligibility criteria and was charted in the evidence map.
73	PMID:30730385	Report assessed	Included	Met the prespecified eligibility criteria and was charted in the evidence map.
74	PMID:29776724	Report assessed	Included	Met the prespecified eligibility criteria and was charted in the evidence map.
75	PMID:28968312	Report assessed	Included	Met the prespecified eligibility criteria and was charted in the evidence map.
76	PMID:28719993	Report assessed	Included	Met the prespecified eligibility criteria and was charted in the evidence map.
77	PMID:28465015	Report assessed	Included	Met the prespecified eligibility criteria and was charted in the evidence map.
78	PMID:28196434	Title/abstract	Excluded at title/abstract	Final consensus exclusion at title/abstract stage; a more granular record-level reason was not retained.
79	PMID:18548355	Report assessed	Included	Met the prespecified eligibility criteria and was charted in the evidence map.
80	PMID:18157050	Report assessed	Excluded after report assessment	Chronic reconstruction/tenodermodesis

No.	Canonical ID	Stage	Final consensus decision	Reason
81	PMID:18090832	Report assessed	Excluded after report assessment	Chronic reconstruction technique
82	PMID:11418917	Report assessed	Excluded after report assessment	Congenital Dupuytren/non-traumatic condition
83	PMID:11077860	Title/abstract	Excluded at title/abstract	Final consensus exclusion at title/abstract stage; a more granular record-level reason was not retained.
84	PMID:1573000	Report assessed	Excluded after report assessment	Chronic reconstruction
85	DOI:10.1097/bpo.0000000000001676	Title/abstract	Excluded at title/abstract	Final consensus exclusion at title/abstract stage; a more granular record-level reason was not retained.
86	DOI:10.1097/bpo.0000000000001632	Title/abstract	Excluded at title/abstract	Final consensus exclusion at title/abstract stage; a more granular record-level reason was not retained.
87	DOI:10.1007/978-3-319-29980-8_60	Title/abstract	Excluded at title/abstract	Final consensus exclusion at title/abstract stage; a more granular record-level reason was not retained.
88	DOI:10.1016/j.ajem.2017.02.015	Title/abstract	Excluded at title/abstract	Final consensus exclusion at title/abstract stage; a more granular record-level reason was not retained.
89	DOI:10.1097/bto.0b013e3181b58eb2	Title/abstract	Excluded at title/abstract	Final consensus exclusion at title/abstract stage; a more granular record-level reason was not retained.
90	DOI:10.1136/bcr-2021-244985	Title/abstract	Excluded at title/abstract	Final consensus exclusion at title/abstract stage; a more granular record-level reason was not retained.

No.	Canonical ID	Stage	Final consensus decision	Reason
91	DOI:10.1007/978-3-319-29980-8_57	Title/abstract	Excluded at title/abstract	Final consensus exclusion at title/abstract stage; a more granular record-level reason was not retained.
92	DOI:10.1016/j.nurpra.2020.06.017	Title/abstract	Excluded at title/abstract	Final consensus exclusion at title/abstract stage; a more granular record-level reason was not retained.
93	DOI:10.29315/gm.v5i2.165	Title/abstract	Excluded at title/abstract	Final consensus exclusion at title/abstract stage; a more granular record-level reason was not retained.
94	DOI:10.1136/bmj.328.7444.900-c	Title/abstract	Excluded at title/abstract	Final consensus exclusion at title/abstract stage; a more granular record-level reason was not retained.
95	DOI:10.1007/978-3-319-56651-1_10	Title/abstract	Excluded at title/abstract	Final consensus exclusion at title/abstract stage; a more granular record-level reason was not retained.
96	DOI:10.1007/978-1-4614-8515-5_44	Title/abstract	Excluded at title/abstract	Final consensus exclusion at title/abstract stage; a more granular record-level reason was not retained.
97	DOI:10.1007/978-1-4614-8758-6_44-1	Title/abstract	Excluded at title/abstract	Final consensus exclusion at title/abstract stage; a more granular record-level reason was not retained.
98	DOI:10.1016/j.visj.2021.100980	Title/abstract	Excluded at title/abstract	Final consensus exclusion at title/abstract stage; a more granular record-level reason was not retained.
99	https://openalex.org/W3019939436	Title/abstract	Excluded at title/abstract	Final consensus exclusion at title/abstract stage; a more granular record-level reason was not retained.
100	DOI:10.1093/bjs/znab259.946	Title/abstract	Excluded at title/abstract	Final consensus exclusion at title/abstract stage; a more granular record-level reason was not retained.

No.	Canonical ID	Stage	Final consensus decision	Reason
101	DOI:10.2106/jbjs.st.24.00024	Title/abstract	Excluded at title/abstract	Final consensus exclusion at title/abstract stage; a more granular record-level reason was not retained.
102	DOI:10.1007/978-3-031-32072-9_6	Title/abstract	Excluded at title/abstract	Final consensus exclusion at title/abstract stage; a more granular record-level reason was not retained.
103	DOI:10.1007/978-3-319-28226-8_60-1	Title/abstract	Excluded at title/abstract	Final consensus exclusion at title/abstract stage; a more granular record-level reason was not retained.
104	DOI:10.1016/j.jpeds.2021.01.056	Title/abstract	Excluded at title/abstract	Final consensus exclusion at title/abstract stage; a more granular record-level reason was not retained.
105	DOI:10.1016/j.jhsa.2014.06.109	Title/abstract	Excluded at title/abstract	Final consensus exclusion at title/abstract stage; a more granular record-level reason was not retained.
106	DOI:10.21203/rs.3.rs-21070/v2	Title/abstract	Excluded at title/abstract	Final consensus exclusion at title/abstract stage; a more granular record-level reason was not retained.
107	DOI:10.1097/bpo.0000000000002820	Title/abstract	Excluded at title/abstract	Final consensus exclusion at title/abstract stage; a more granular record-level reason was not retained.
108	DOI:10.1016/j.joscr.2023.09.007	Report assessed	Included	Met the prespecified eligibility criteria and was charted in the evidence map.
109	DOI:10.21203/rs.3.rs-21070/v1	Title/abstract	Excluded at title/abstract	Final consensus exclusion at title/abstract stage; a more granular record-level reason was not retained.
110	DOI:10.1016/j.joscr.2024.12.001	Report assessed	Included	Met the prespecified eligibility criteria and was charted in the evidence map.

No.	Canonical ID	Stage	Final consensus decision	Reason
111	DOI:10.1017/cem.2020.293	Title/abstract	Excluded at title/abstract	Final consensus exclusion at title/abstract stage; a more granular record-level reason was not retained.
112	DOI:10.4103/atr.atr_9_22	Report assessed	Included	Met the prespecified eligibility criteria and was charted in the evidence map.
113	DOI:10.1016/j.jhsa.2013.09.011	Title/abstract	Excluded at title/abstract	Final consensus exclusion at title/abstract stage; a more granular record-level reason was not retained.
114	DOI:10.1055/b-0040-177446	Title/abstract	Excluded at title/abstract	Final consensus exclusion at title/abstract stage; a more granular record-level reason was not retained.
115	DOI:10.1007/978-981-10-4400-7_22	Title/abstract	Excluded at title/abstract	Final consensus exclusion at title/abstract stage; a more granular record-level reason was not retained.
116	DOI:10.1007/978-3-319-28226-8_57-1	Title/abstract	Excluded at title/abstract	Final consensus exclusion at title/abstract stage; a more granular record-level reason was not retained.
117	DOI:10.1542/pir.2015-0160	Title/abstract	Excluded at title/abstract	Final consensus exclusion at title/abstract stage; a more granular record-level reason was not retained.
118	DOI:10.1302/2048-0105.121.3600028	Title/abstract	Excluded at title/abstract	Final consensus exclusion at title/abstract stage; a more granular record-level reason was not retained.
119	DOI:10.1007/s12178-017-9395-6	Title/abstract	Excluded at title/abstract	Final consensus exclusion at title/abstract stage; a more granular record-level reason was not retained.
120	DOI:10.1542/pir.36.5.184	Title/abstract	Excluded at title/abstract	Final consensus exclusion at title/abstract stage; a more granular record-level reason was not retained.

No.	Canonical ID	Stage	Final consensus decision	Reason
121	DOI:10.1542/pir.2021-004987	Title/abstract	Excluded at title/abstract	Final consensus exclusion at title/abstract stage; a more granular record-level reason was not retained.
122	DOI:10.1007/s12178-012-9146-7	Title/abstract	Excluded at title/abstract	Final consensus exclusion at title/abstract stage; a more granular record-level reason was not retained.
123	DOI:10.62547/rurz7083	Title/abstract	Excluded at title/abstract	Final consensus exclusion at title/abstract stage; a more granular record-level reason was not retained.
124	DOI:10.3390/children10091446	Title/abstract	Excluded at title/abstract	Final consensus exclusion at title/abstract stage; a more granular record-level reason was not retained.
125	DOI:10.5772/intechopen.76658	Title/abstract	Excluded at title/abstract	Final consensus exclusion at title/abstract stage; a more granular record-level reason was not retained.
126	DOI:10.1055/s-0037-1602414	Title/abstract	Excluded at title/abstract	Final consensus exclusion at title/abstract stage; a more granular record-level reason was not retained.
127	DOI:10.1007/s00264-023-05858-y	Title/abstract	Excluded at title/abstract	Final consensus exclusion at title/abstract stage; a more granular record-level reason was not retained.
128	DOI:10.25214/28056272.1180	Title/abstract	Excluded at title/abstract	Final consensus exclusion at title/abstract stage; a more granular record-level reason was not retained.
129	DOI:10.52312/jdrs.2023.994	Title/abstract	Excluded at title/abstract	Final consensus exclusion at title/abstract stage; a more granular record-level reason was not retained.
130	DOI:10.7759/cureus.17246	Title/abstract	Excluded at title/abstract	Final consensus exclusion at title/abstract stage; a more granular record-level reason was not retained.

No.	Canonical ID	Stage	Final consensus decision	Reason
131	DOI:10.1007/978-3-319-29980-8	Title/abstract	Excluded at title/abstract	Final consensus exclusion at title/abstract stage; a more granular record-level reason was not retained.
132	DOI:10.3389/fped.2023.1118954	Title/abstract	Excluded at title/abstract	Final consensus exclusion at title/abstract stage; a more granular record-level reason was not retained.
133	DOI:10.46747/cfp.720293	Title/abstract	Excluded at title/abstract	Final consensus exclusion at title/abstract stage; a more granular record-level reason was not retained.
134	DOI:10.52965/001c.125898	Title/abstract	Excluded at title/abstract	Final consensus exclusion at title/abstract stage; a more granular record-level reason was not retained.
135	DOI:10.13112/pc.442	Title/abstract	Excluded at title/abstract	Final consensus exclusion at title/abstract stage; a more granular record-level reason was not retained.
136	DOI:10.1007/978-3-031-32072-9	Title/abstract	Excluded at title/abstract	Final consensus exclusion at title/abstract stage; a more granular record-level reason was not retained.
137	DOI:10.52581/1814-1471/93/10	Title/abstract	Excluded at title/abstract	Final consensus exclusion at title/abstract stage; a more granular record-level reason was not retained.
138	DOI:10.46747/cfp.6909615	Title/abstract	Excluded at title/abstract	Final consensus exclusion at title/abstract stage; a more granular record-level reason was not retained.
139	DOI:10.1016/j.jposna.2026.100365	Title/abstract	Excluded at title/abstract	Final consensus exclusion at title/abstract stage; a more granular record-level reason was not retained.
140	DOI:10.18019/1028-4427-2021-27-3-322-324	Title/abstract	Excluded at title/abstract	Final consensus exclusion at title/abstract stage; a more granular record-level reason was not retained.

No.	Canonical ID	Stage	Final consensus decision	Reason
141	DOI:10.7759/cureus.87790	Title/abstract	Excluded at title/abstract	Final consensus exclusion at title/abstract stage; a more granular record-level reason was not retained.
142	DOI:10.18019/1028-4427-2021-27-3-319-321	Title/abstract	Excluded at title/abstract	Final consensus exclusion at title/abstract stage; a more granular record-level reason was not retained.
143	DOI:10.52965/001c.125161	Title/abstract	Excluded at title/abstract	Final consensus exclusion at title/abstract stage; a more granular record-level reason was not retained.
144	DOI:10.52783/jes.8388	Title/abstract	Excluded at title/abstract	Final consensus exclusion at title/abstract stage; a more granular record-level reason was not retained.
145	DOI:10.7759/cureus.104513	Title/abstract	Excluded at title/abstract	Final consensus exclusion at title/abstract stage; a more granular record-level reason was not retained.
146	DOI:10.1016/j.jhsa.2026.04.010	Title/abstract	Excluded at title/abstract	Final consensus exclusion at title/abstract stage; a more granular record-level reason was not retained.
147	DOI:10.52312/jdrs.2026.2698	Title/abstract	Excluded at title/abstract	Final consensus exclusion at title/abstract stage; a more granular record-level reason was not retained.
148	DOI:10.14292/totbid.dergisi.2019.51	Title/abstract	Excluded at title/abstract	Final consensus exclusion at title/abstract stage; a more granular record-level reason was not retained.
149	DOI:10.46747/cfp.7202e30	Title/abstract	Excluded at title/abstract	Final consensus exclusion at title/abstract stage; a more granular record-level reason was not retained.
150	DOI:10.37811/cl_rcm.v9i2.17176	Title/abstract	Excluded at title/abstract	Final consensus exclusion at title/abstract stage; a more granular record-level reason was not retained.

No.	Canonical ID	Stage	Final consensus decision	Reason
151	DOI:10.13107/jocr.2023.v13.i11.4014	Title/abstract	Excluded at title/abstract	Final consensus exclusion at title/abstract stage; a more granular record-level reason was not retained.
152	DOI:10.1007/978-3-032-17324-9	Title/abstract	Excluded at title/abstract	Final consensus exclusion at title/abstract stage; a more granular record-level reason was not retained.
153	DOI:10.14292/totbid.dergisi.2014.13	Title/abstract	Excluded at title/abstract	Final consensus exclusion at title/abstract stage; a more granular record-level reason was not retained.
154	DOI:10.5578/totbid.dergisi.2024.36	Title/abstract	Excluded at title/abstract	Final consensus exclusion at title/abstract stage; a more granular record-level reason was not retained.
155	DOI:10.35631/ijepc.1163033	Title/abstract	Excluded at title/abstract	Final consensus exclusion at title/abstract stage; a more granular record-level reason was not retained.
156	DOI:10.4103/tjps.tjps_85_25	Report assessed	Included	Met the prespecified eligibility criteria and was charted in the evidence map.
157	DOI:10.1542/peds.141.1_meetingabstract.665	Title/abstract	Excluded at title/abstract	Final consensus exclusion at title/abstract stage; a more granular record-level reason was not retained.
158	DOI:10.1542/peds.141.1ma7.665	Title/abstract	Excluded at title/abstract	Final consensus exclusion at title/abstract stage; a more granular record-level reason was not retained.
159	DOI:10.29245/2767-5130/2025/2.1221	Report assessed	Included	Met the prespecified eligibility criteria and was charted in the evidence map.
160	DOI:10.47363/jsar/2025(6)212	Report assessed	Included	Met the prespecified eligibility criteria and was charted in the evidence map.

No.	Canonical ID	Stage	Final consensus decision	Reason
161	DOI:10.7197/cmj.v37i2.5000079536	Report assessed	Included	Met the prespecified eligibility criteria and was charted in the evidence map.

S2.4. Report-level and other-method decisions

Database reports excluded after assessment (n=26).

Report	Reason
PMID 32576259	Protocol without results
PMID 35489831	Reprint/duplicate publication
PMID 34104672	Adult population
PMID 24194615	Adult population
PMID 31690204	Adult case (24 years)
PMID 18157050	Chronic reconstruction/tenodermodesis
PMID 18090832	Chronic reconstruction technique
PMID 1573000	Chronic reconstruction
PMID 30808392	Chronic reconstruction
PMID 39311252	Chronic reconstruction; population outside target
PMID 40073800	Non-traumatic osteochondroma
PMID 11418917	Congenital Dupuytren/non-traumatic condition
PMID 9620197	Repetitive overuse rather than target acute trauma
PMID 9763283	Bibliographic duplicate of PMID 9620197
PMID 41253575	Mixed 3D-splint review without separable pediatric target result
PMID 30759479	Broad phalangeal cohort without separable target result
PMID 37020613	Broad hand cohort without separable target result
PMID 40838653	Clinical image without extractable data
PMID 40040758	Routine single case without a distinct phenotype/complication
PMID 34557438	Routine single case without a distinct phenotype/complication
PMID 33133852	Routine single case without a distinct phenotype/complication
PMID 24841396	Clinical image without extractable data
PMID 41106872	Multi-trauma review without extractable target synthesis
PMID 37704235	General nail-trauma review without target result
PMID 32586465	General hand-infection review without target cohort

Report	Reason
PMID 31103087	General hand/wrist-fracture review without separable target synthesis

Other-method reports excluded (n=6).

Report	Reason
Preprints DOI 10.20944/preprints202406.0990.v1	Prepublication not peer reviewed; evidence awaiting peer review
Jain et al., NINJA RCT	Outside mapped concept; fractures requiring fixation were excluded and no Seymour subgroup
BSSH Mallet Injuries standard	External clinical triangulation, not an included research source
RCH Phalangeal Finger Fractures guideline	External clinical triangulation, not an included research source
BSSH Open Fractures Other Than Tuft Fractures standard	External clinical triangulation, not an included research source
RCH Fingertip and Nail Injuries guideline	External clinical triangulation, not an included research source

Other-method reports included (n=9).

Report	Decision	Reason
PMID:5939484	Included	Met eligibility criteria and was charted in S2.1.
PMID:681400	Included	Met eligibility criteria and was charted in S2.1.
PMID:1624869	Included	Met eligibility criteria and was charted in S2.1.
PMID:8349960	Included	Met eligibility criteria and was charted in S2.1.
PMID:7797986	Included	Met eligibility criteria and was charted in S2.1.
PMID:10357547	Included	Met eligibility criteria and was charted in S2.1.
PMID:11386767	Included	Met eligibility criteria and was charted in S2.1.
PMID:20724203	Included	Met eligibility criteria and was charted in S2.1.
PMID:24209954	Included	Met eligibility criteria and was charted in S2.1.

S2.5. Included bibliography (exactly 60 sources)

1. Seymour N. Juxta-epiphysial fracture of the terminal phalanx of the finger. J Bone Joint Surg Br. 1966;48(2):347-9. doi:10.1302/0301-620X.48B2.347.
2. Engber WD, Clancy WG. Traumatic avulsion of the finger nail associated with injury to the phalangeal epiphyseal plate. J Bone Joint Surg Am. 1978;60(5):713-4.
3. Barton NJ. Fractures of the phalanges of the hand in children. Hand. 1979;11(2):134-43. doi:10.1016/s0072-968x(79)80025-x.
4. Michelinakis E, Vourexaki H. Displaced epiphyseal plate of the terminal phalanx in a child. Hand. 1980;12(1):51-3. doi:10.1016/s0072-968x(80)80029-5.
5. Masquelet AC, Romana C. [Infection of the tendon sheath following an open epiphyseal fracture. Apropos of a case]. Ann Chir Main. 1988;7(1):72-4. doi:10.1016/s0753-9053(88)80072-3.
6. Banerjee A. Irreducible distal phalangeal epiphyseal injuries. J Hand Surg Br. 1992;17(3):337-8. doi:10.1016/0266-7681(92)90124-k.
7. Waters PM, Benson LS. Dislocation of the distal phalanx epiphysis in toddlers. J Hand Surg Am. 1993;18(4):581-585. doi:10.1016/0363-5023(93)90293-C.
8. Inglefield CJ, D'Arcangelo M, Kolhe PS. Injuries to the nail bed in childhood. J Hand Surg Br. 1995;20(2):258-61. doi:10.1016/s0266-7681(05)80066-x.
9. Nissenbaum M. Avascular necrosis of the distal phalangeal epiphysis following physeal fracture: a case report. J Hand Surg Am. 1999;24(3):633-637. doi:10.1053/jhsu.1999.0633.
10. Al-Qattan MM. Extra-articular transverse fractures of the base of the distal phalanx (Seymour's fracture) in children and adults. J Hand Surg Br. 2001;26(3):201-6. doi:10.1054/jhsb.2000.0549.
11. Ganayem M, Edelson G. Base of distal phalanx fracture in children: a mallet finger mimic. J Pediatr Orthop. 2005;25(4):487-9. doi:10.1097/01.bpo.0000158813.37225.fa.
12. Schmidt B, Weinberg A, Friedrich H. [The mallet finger in children and adolescents]. Handchir Mikrochir Plast Chir. 2008;40(3):149-52. doi:10.1055/s-2007-965475.
13. Zipoli B, Donon L, Ribeyre D. [Surf and Seymour's fractures]. Chir Main. 2010;29(4):263-265. doi:10.1016/j.main.2010.06.010.
14. Abzug JM, Kozin SH. Seymour fractures. J Hand Surg Am. 2013;38(11):2267-70; quiz 2270. doi:10.1016/j.jhsa.2013.08.104.
15. Krusche-Mandl I, Köttstorfer J, Thalhammer G, Aldrian S, Erhart J, Platzer P. Seymour fractures: retrospective analysis and therapeutic considerations. J Hand Surg Am. 2013;38(2):258-64. doi:10.1016/j.jhsa.2012.11.015.
16. Nellans KW, Chung KC. Pediatric hand fractures. Hand Clin. 2013;29(4):569-578. doi:10.1016/j.hcl.2013.08.009.
17. Han C, Naito K, Sugiyama Y, Obayashi O, Kaneko K. Bony mallet finger without epiphyseal plate injury in childhood. Int J Surg Case Rep. 2015;14:172-4. doi:10.1016/j.ijscr.2015.08.005.
18. Taş T, Kılınç S, Güneyli V, Bulut O, Çilli K, Zengin E. Assesment of conservative therapy for pediatric patients with acute mallet finger deformity. Cumhuriyet Med J. 2015;37(2):121-125. doi:10.7197/cmj.v37i2.5000079536.
19. Goodell PB, Bauer A. Problematic Pediatric Hand and Wrist Fractures. JBJS Rev. 2016;4(5). doi:10.2106/JBJS.RVW.O.00028.
20. Zhang L, Zuo Y, Wang Y, Zhou H, Wang G, Yu T, et al. [SURGICAL TREATMENT OF Seymour FRACTURES IN CHILDREN AND ADOLESCENTS]. Zhongguo Xiu Fu Chong Jian Wai Ke Za Zhi. 2016;30(5):529-531. doi:10.7507/1002-1892.20160106.
21. Forward KE, Yazdani A, Lim R. Mallet Finger in a Toddler: A Rare But Easily Missed Injury. Pediatr Emerg Care. 2017;33(10):e103-e104. doi:10.1097/PEC.0000000000001268.
22. Kattan AE, AlShomer F, Alhujayri AK, Alfowzan M, Murrad KA, Alsajjan H. A case series of pediatric seymour fractures related to hoverboards: Increasing trend with changing lifestyle. Int J Surg Case Rep. 2017;38:57-60. doi:10.1016/j.ijscr.2017.06.058.
23. Lankachandra M, Wells CR, Cheng CJ, Hutchison RL. Complications of Distal Phalanx Fractures in Children. J Hand Surg Am. 2017;42(7):574.e1-574.e6. doi:10.1016/j.jhsa.2017.03.042.
24. Reyes BA, Ho CA. The High Risk of Infection With Delayed Treatment of Open Seymour Fractures: Salter-Harris I/II or Juxta-epiphyseal Fractures of the Distal Phalanx With Associated Nailbed Laceration. J Pediatr Orthop. 2017;37(4):247-253. doi:10.1097/BPO.0000000000000638.
25. Chen AT, Conry KT, Gilmore A, Son-Hing JP, Liu RW. Outcomes Following Operative Treatment of Adolescent Mallet Fractures. HSS J. 2018;14(1):83-87. doi:10.1007/s11420-017-9563-7.
26. Lin JS, Samora JB. Outcomes of Splinting in Pediatric Mallet Finger. J Hand Surg Am. 2018;43(11):1041.e1-1041.e9. doi:10.1016/j.jhsa.2018.03.037.
27. Ota T, Itoh S, Matsuyama Y. Comparison of Treatment Results for Mallet Finger Fractures in Children Between Low-Intensity Pulsed Ultrasound Stimulation and Ishiguro's Method. Hand (N Y). 2018;13(1):80-85. doi:10.1177/1558944717692095.
28. Lin JS, Popp JE, Balch Samora J. Treatment of Acute Seymour Fractures. J Pediatr Orthop. 2019;39(1):e23-e27. doi:10.1097/BPO.0000000000001275.
29. Reddy M, Ho CA. Comparison of Percutaneous Reduction and Pin Fixation in Acute and Chronic Pediatric Mallet Fractures. J Pediatr Orthop. 2019;39(3):146-152. doi:10.1097/BPO.0000000000000896.
30. Cha SM, Kang JW, Shin HD, Ga IH. Acute Seymour fractures in children/adolescents overlooked as a simple nail injury: Reduction without pinning. Hand Surg Rehabil. 2021;40(2):171-176. doi:10.1016/j.hansur.2020.11.009.
31. McVeigh KH, Berger TG, Cudahy R, Dekker TM, Brigham TJ, Braxton JC. An Evidence-Based Approach to Casting and Orthosis Management of the Pediatric, Adolescent, and Young Adult Population for Injuries of the Upper Extremity: A Review Article. Clin J Sport Med. 2021;31(2):151-162. doi:10.1097/JSM.0000000000000718.

32. Perez-Lopez LM, Parada-Avendaño I, Cabrera-Gonzalez M, Fontecha CG. Seymour fracture: Better do not underestimate it. Jt Dis Relat Surg. 2021;32(3):569-574. doi:10.52312/jdrs.2021.330.
33. Rask DMG, Wingfield J, Elrick B, Chen C, Lalka A, Sibbel SE, et al. Seymour Fractures: A Retrospective Review of Infection Rates, Treatment and Timing of Antibiotic Administration. Pediatr Emerg Care. 2021;37(12):e1299-e1302. doi:10.1097/PEC.0000000000002009.
34. Samade R, Lin JS, Popp JE, Samora JB. Delayed Presentation of Seymour Fractures: A Single Institution Experience and Management Recommendations. Hand (N Y). 2021;16(5):686-693. doi:10.1177/1558944719878846.
35. Afshar A, Tabrizi A, Taleb H. Bipartite nail deformity due to Seymour's fracture in a pediatric patient. Arch Trauma Res. 2022;11(2):101-103. doi:10.4103/atr.atr_9_22.
36. Cha SM, Lin YT. Treatment Strategies for Acute Seymour Fractures in Children and Adolescents: Including Crushed Open Fractures. Ann Plast Surg. 2022;89(4):380-384. doi:10.1097/SAP.0000000000003261.
37. Houlden R. Does K-wire fixation improve outcomes in children with a Seymour fracture? Ann Med Surg (Lond). 2022;82:104566. doi:10.1016/j.amsu.2022.104566.
38. Kiely AL, Nolan GS, Cooper LRL. Management of Seymour Fractures in Children and Adolescents: A Systematic Review and Meta-Analysis. J Hand Surg Am. 2022;47(12):1223.e1-1223.e20. doi:10.1016/j.jhsa.2021.08.022.
39. Testa EJ, Marcaccio SE, Kosinski LR, Jones MC, Katarincic JA. Salter-Harris Type III Fracture of the Distal Phalanx: A Rare Juxtaphyseal Variant. Hand (N Y). 2022;17(6):NP6-NP10. doi:10.1177/15589447221082165.
40. Albahlal JM, Alzahrani A, Khan H. Multiple Bony Mallet Finger Injuries in One Hand of a 14-Year-Old Boy. Cureus. 2023;15(8):e44441. doi:10.7759/cureus.44441.
41. Dibbs RP, Mitchell TW, Baumgartner RE, Koshy JC, Bell BR. Complication Rates in Patients With Classic and Radiographic Variants of Seymour Fractures. J Pediatr Orthop. 2023;43(2):e157-e162. doi:10.1097/BPO.0000000000002306.
42. Englert EG, Tooley T, Weisz K, Shapiro P. Suture Fixation of Subacute Pediatric Seymour Fractures. J Hand Surg Glob Online. 2023;5(2):231-233. doi:10.1016/j.jhsg.2022.10.013.
43. Millar WA, Marchessault JA. Mallet Finger Autofusion in Adolescent Post-Pin Fixation Surgery - A Case Report. J Orthop Case Rep. 2023;13(5):116-119. doi:10.13107/jocr.2023.v13.i05.3670.
44. Yamamoto K, Shimura H, Suzuki H, Yamada E, Fujita K, Nimura A. Acute osteomyelitis after the physeal injury of the distal phalanx of the hand (Seymour fracture): a report of two cases. JOS Case Rep. 2023;2(4):165-168. doi:10.1016/j.joscr.2023.09.007.
45. Acciaro AL, Gravina D, Pantaleoni F, Cataldo G, Adani R. Retrospective study of Ishiguro's technique for mallet bone finger in children: long-term follow-up and analysis of predictors in outcomes. Int Orthop. 2024;48(6):1501-1506. doi:10.1007/s00264-024-06162-z.
46. Jirlow U, Aydin U. [The Seymour fracture - a fracture that easily can be missed]. Lakartidningen. 2024;121.
47. Baur A, Anthony T, Lustig K, Lee ML. Open Reduction and Internal Fixation of a Volar Displaced Salter-Harris III Mallet Fracture in a Pediatric Patient: A Case Report. Pediatr Rep. 2025;17(4):82. doi:10.3390/pediatric17040082.
48. El Sewify O, Bouhadana G, Lorange E, Sylvain M, Nordback PH, Das De S, et al. A systematic review of surgical management for open fractures of the distal phalanx: What is the evidence? J Hand Microsurg. 2025;17(5):100318. doi:10.1016/j.jham.2025.100318.
49. Gottschalk HP, McMahon ND, Piper K, Lawson K, Bauer A, Chaundhry S, et al. It's Just a Fingertip! Yet Controversy Exists: Standardizing a Treatment Pathway for Pediatric Fingertip Injuries. J Pediatr Soc North Am. 2025;11:100163. doi:10.1016/j.jposna.2025.100163.
50. Gravina D, Portoghese A, Pantaleoni F, Montanari M, Manfredi A, Acciaro AL. Bony mallet finger: a comparative review of approaches in pediatric vs adult populations. J Orthop Orthop Surg. 2025;6(2):8-12. doi:10.29245/2767-5130/2025/2.1221.
51. Hosozawa K, Shibuya R, Miyamura S, Iwahashi T, Fuji T. Two cases of treatment of the Seymour fracture complicated by pyogenic arthritis with antibiotic-impregnated calcium phosphate paste granules. JOS Case Rep. 2025;4(2):63-66. doi:10.1016/j.joscr.2024.12.001.
52. Jose Jerome JT. Management of Seymour (Distal Phalangeal) Fractures with Nail Bed Involvement. JBJS Essent Surg Tech. 2025;15(3). doi:10.2106/JBJS.ST.24.00041.
53. Odorizzi M, Odorizzi G. Treatment of pediatric bone mallet finger: the current state of the art and a proposal of algorithm treatment. J Surg Anesth Res. 2025;6(4):1-4. doi:10.47363/JSAR/2025(6)212.
54. Rubin G, Eliyahu AH, Mano UD, Shay R, Svorai S, Sagiv R, et al. The outcome difference between acute bony and tendinous mallet fingers treated conservatively in children. J Hand Ther. 2025;38(1):68-72. doi:10.1016/j.jht.2024.07.004.
55. Yiğitbay A, Yıldırım G, Ari MC, Çetin H, Kural C. The silent threat under the nail: Evaluation of Seymour fractures in pediatric patients. Jt Dis Relat Surg. 2025;36(2):320-327. doi:10.52312/jdrs.2025.2066.
56. Conroy JL, Dunham AM, Stepanovich M, Herman MJ, Abzug JM. Physeal Phalanx Fractures: To Fix or Not To Fix? Instr Course Lect. 2026;75:625-638.
57. Lalchandani GR, Berger A, Schroeder NS, Bauer AS. Pediatric Finger Fractures: Preventing Big Problems After Small Fractures. J Hand Surg Am. 2026;51(3):324-333. doi:10.1016/j.jhsa.2025.08.015.

58. Ross SJ, Lu S, Engel AH, Doyle JS, Ferguson S, Doyle SM. Seymour Fractures Revisited: Recognition and Management of an Open Physeal Distal Phalanx Injury. J Pediatr Soc North Am. 2026;16:100404. doi:10.1016/j.jposna.2026.100404.
59. Sinclair MK, Santana D, Manske MC. Management of Pediatric Hand Injuries in the Acute Setting Part 2: Fractures and Dislocations. J Pediatr Soc North Am. 2026;15:100364. doi:10.1016/j.jposna.2026.100364.
60. Yildiran G, Oguz M, Akdeniz H, Tosun Z. Clinical and Radiological Outcomes of Surgically Treated Pediatric Mallet Finger. Turk J Plast Surg. 2026;34(2):63-67. Available online: https://www.ovid.com/jnls/tjps/fulltext/10.4103/tjps.tjps_85_25~clinical-and-radiological-outcomes-of-surgically-treated (accessed on 19 September 2026).

S2.6. Deduplication audit (58 removed occurrences)

The log below records 10 within-PubMed, two within-OpenAlex, and 46 cross-source removed occurrences. Canonical retention favored PubMed when a DOI or normalized title matched an OpenAlex work. A machine-readable version accompanies the review audit trail.

No.	Level	Removed occurrence	Title	Retained ID	Match basis
1	within PubMed	PubMed B | PMID:40861033	Management of Seymour (Distal Phalangeal) Fractures with Nail Bed Involvement.	PMID:40861033	identical PMID
2	within PubMed	PubMed B | PMID:34557438	Seymour's Fracture in a 13-years-old Child: A Case Report.	PMID:34557438	identical PMID
3	within PubMed	PubMed B | PMID:33133852	Seymour Fracture in a Pediatric Patient: A Case Report.	PMID:33133852	identical PMID
4	within PubMed	PubMed B | PMID:30759479	The patterns of phalangeal fractures in children and adolescents: a review of 512 cases.	PMID:30759479	identical PMID
5	within PubMed	PubMed B | PMID:29399000	Outcomes Following Operative Treatment of Adolescent Mallet Fractures.	PMID:29399000	identical PMID
6	within PubMed	PubMed B | PMID:27490217	Problematic Pediatric Hand and Wrist Fractures.	PMID:27490217	identical PMID
7	within PubMed	PubMed B | PMID:26279261	Bony mallet finger without epiphyseal plate injury in childhood.	PMID:26279261	identical PMID
8	within PubMed	PubMed B | PMID:24194615	Distal phalanx fracture in adults: Seymour-type fracture.	PMID:24194615	identical PMID
9	within PubMed	PubMed B | PMID:15958901	Base of distal phalanx fracture in children: a mallet finger mimic.	PMID:15958901	identical PMID
10	within PubMed	PubMed B | PMID:7364328	Displaced epiphyseal plate of the terminal phalanx in a child.	PMID:7364328	identical PMID
11	within OpenAlex	OpenAlex Seymour full-text | https://openalex.org/W3012054534	Seymour Fractures: A Retrospective Review of Infection Rates, Treatment and Timing of Antibiotic Administration.	https://openalex.org/W4288066231	identical DOI
12	within OpenAlex	OpenAlex pediatric mallet title | https://openalex.org/W4385267520	Seymour Fractures and Bony Mallet Fractures in the Pediatric Population	https://openalex.org/W4385267520	identical DOI
13	cross-source	OpenAlex Seymour full-text | https://openalex.org/W2915036642	Seymour Fractures	PMID:24206995	identical DOI
14	cross-source	OpenAlex Seymour full-text | https://openalex.org/W2038814071	Seymour Fractures: Retrospective Analysis and Therapeutic Considerations	PMID:23351909	identical DOI

No.	Level	Removed occurrence	Title	Retained ID	Match basis
15	cross-source	OpenAlex Seymour full-text | https://openalex.org/W2898429059	Treatment of Acute Seymour Fractures	PMID:30358692	identical DOI
16	cross-source	OpenAlex Seymour full-text; OpenAlex Seymour full-text | https://openalex.org/W4288066231	Seymour Fractures	PMID:32149995	identical DOI
17	cross-source	OpenAlex Seymour full-text | https://openalex.org/W2319179232	The High Risk of Infection With Delayed Treatment of Open Seymour Fractures: Salter-Harris I/II or Juxta-epiphyseal Fractures of the Distal Phalanx With Associated Nailbed Laceration	PMID:26327401	identical DOI
18	cross-source	OpenAlex Seymour full-text | https://openalex.org/W2736043735	A case series of pediatric seymour fractures related to hoverboards	PMID:28735119	identical DOI
19	cross-source	OpenAlex Seymour full-text | https://openalex.org/W3215895348	Management of Seymour Fractures in Children and Adolescents: A Systematic Review and Meta-Analysis	PMID:34810026	identical DOI
20	cross-source	OpenAlex Seymour full-text | https://openalex.org/W3216497439	Seymour fracture: Better do not underestimate it	PMID:34842086	identical DOI
21	cross-source	OpenAlex Seymour full-text | https://openalex.org/W2979734935	Delayed Presentation of Seymour Fractures: A Single Institution Experience and Management Recommendations	PMID:31597480	identical DOI
22	cross-source	OpenAlex Seymour full-text | https://openalex.org/W2983254451	Open Infected Seymour Fracture	PMID:31690204	identical DOI
23	cross-source	OpenAlex Seymour full-text | https://openalex.org/W3037960422	The optimal management of Seymour fractures in children and adolescents: a systematic review protocol	PMID:32576259	identical DOI
24	cross-source	OpenAlex Seymour full-text | https://openalex.org/W2945440254	Pediatric Hand and Wrist Fractures	PMID:31103087	identical DOI
25	cross-source	OpenAlex Seymour full-text | https://openalex.org/W4296901257	Does K-wire fixation improve outcomes in children with a Seymour fracture?	PMID:36268384	identical DOI

No.	Level	Removed occurrence	Title	Retained ID	Match basis
26	cross-source	OpenAlex Seymour full-text | https://openalex.org/W4313624870	Complication Rates in Patients With Classic and Radiographic Variants of Seymour Fractures	PMID:36607925	identical DOI
27	cross-source	OpenAlex Seymour full-text | https://openalex.org/W3121252522	Acute Seymour fractures in children/adolescents overlooked as a simple nail injury: Reduction without pinning	PMID:33508522	identical DOI
28	cross-source	OpenAlex Seymour full-text | https://openalex.org/W3088102538	Seymour Fracture in a Pediatric Patient: A Case Report	PMID:33133852	identical DOI
29	cross-source	OpenAlex Seymour full-text | https://openalex.org/W4282974420	Treatment Strategies for Acute Seymour Fractures in Children and Adolescents	PMID:35703237	identical DOI
30	cross-source	OpenAlex Seymour full-text | https://openalex.org/W2978753155	[SURGICAL TREATMENT OF Seymour FRACTURES IN CHILDREN AND ADOLESCENTS].	PMID:29786288	identical DOI
31	cross-source	OpenAlex Seymour full-text | https://openalex.org/W4310804809	Suture Fixation of Subacute Pediatric Seymour Fractures	PMID:36974281	identical DOI
32	cross-source	OpenAlex Seymour full-text | https://openalex.org/W2345343321	Problematic Pediatric Hand and Wrist Fractures	PMID:27490217	identical DOI
33	cross-source	OpenAlex Seymour full-text | https://openalex.org/W4409234898	The silent threat under the nail: Evaluation of Seymour fractures in pediatric patients	PMID:40235410	identical DOI
34	cross-source	OpenAlex Seymour full-text | https://openalex.org/W2580474543	Musculoskeletal Injuries Resulting from Use of Hoverboards: Safety Concerns With an Unregulated Consumer Product	PMID:28110548	identical DOI
35	cross-source	OpenAlex Seymour full-text | https://openalex.org/W3036129984	Pediatric Hand Infections	PMID:32586465	identical DOI
36	cross-source	OpenAlex Seymour full-text | https://openalex.org/W2912672827	The patterns of phalangeal fractures in children and adolescents: a review of 512 cases	PMID:30759479	identical DOI
37	cross-source	OpenAlex Seymour full-text | https://openalex.org/W4413557863	Minor Trauma that Ends up Being a Major Headache	PMID:41106872	identical DOI

No.	Level	Removed occurrence	Title	Retained ID	Match basis
38	cross-source	OpenAlex Seymour full-text | https://openalex.org/W4362512875	Phalangeal and Metacarpal Fractures in Children: A 10-Year Comparison of Factors Affecting Functional Outcomes in 313 Patients	PMID:37020613	identical DOI
39	cross-source	OpenAlex Seymour full-text | https://openalex.org/W4386715926	Approach to nail trauma for primary care physicians	PMID:37704235	identical DOI
40	cross-source	OpenAlex Seymour full-text | https://openalex.org/W4238309666	Seymour fracture	PMID:40838653	normalized title
41	cross-source	OpenAlex Seymour full-text | https://openalex.org/W4413399549	Seymour fracture	PMID:40838653	identical DOI
42	cross-source	OpenAlex Seymour full-text | https://openalex.org/W4240567694	Seymour fracture	PMID:40838653	normalized title
43	cross-source	OpenAlex Seymour full-text | https://openalex.org/W4415598289	Seymour fracture	PMID:40838653	normalized title
44	cross-source	OpenAlex Seymour full-text | https://openalex.org/W4417152619	Seymour fracture	PMID:40838653	normalized title
45	cross-source	OpenAlex Seymour full-text | https://openalex.org/W4403084480	[The Seymour fracture - a fracture that easily can be missed].	PMID:39354737	normalized title
46	cross-source	OpenAlex Seymour full-text | https://openalex.org/W4225277383	Reprint of: seymour fracture: A particularly tuft diagnosis	PMID:35489831	identical DOI
47	cross-source	OpenAlex Seymour full-text | https://openalex.org/W4408141352	Seymour fracture management and functional outcome assessment: a case report	PMID:40040758	identical DOI
48	cross-source	OpenAlex Seymour full-text | https://openalex.org/W3163175758	A rare case of Seymour fracture in an adult with non-fused growth plates	PMID:34104672	identical DOI
49	cross-source	OpenAlex Seymour full-text | https://openalex.org/W7164556992	Seymour Fractures Revisited: Recognition and Management of an Open Physeal Distal Phalanx Injury	PMID:42434507	identical DOI

No.	Level	Removed occurrence	Title	Retained ID	Match basis
50	cross-source	OpenAlex Seymour full-text | https://openalex.org/W4221110228	Salter-Harris Type III Fracture of the Distal Phalanx: A Rare Juxtaphyseal Variant	PMID:35311365	identical DOI
51	cross-source	OpenAlex Seymour full-text | https://openalex.org/W4413511620	Management of Seymour (Distal Phalangeal) Fractures with Nail Bed Involvement	PMID:40861033	identical DOI
52	cross-source	OpenAlex Seymour full-text | https://openalex.org/W4416738180	Physeal Phalanx Fractures: To Fix or Not To Fix?	PMID:41289484	normalized title
53	cross-source	OpenAlex Seymour full-text | https://openalex.org/W4408312592	It's Just a Fingertip! Yet Controversy Exists: Standardizing a Treatment Pathway for Pediatric Fingertip Injuries	PMID:40432862	identical DOI
54	cross-source	OpenAlex Seymour full-text | https://openalex.org/W3193589009	Seymour's Fracture in a 13-years-old Child: A Case Report	PMID:34557438	identical DOI
55	cross-source	OpenAlex pediatric mallet title | https://openalex.org/W2803157188	Outcomes of Splinting in Pediatric Mallet Finger	PMID:29776724	identical DOI
56	cross-source	OpenAlex pediatric mallet title | https://openalex.org/W2550387502	Comparison of Percutaneous Reduction and Pin Fixation in Acute and Chronic Pediatric Mallet Fractures	PMID:30730419	identical DOI
57	cross-source	OpenAlex pediatric mallet title | https://openalex.org/W4408103635	Finger osteochondroma presenting as non-traumatic mallet finger in pediatric: A case report	PMID:40073800	identical DOI
58	cross-source	OpenAlex pediatric mallet title | https://openalex.org/W4413008293	Open Reduction and Internal Fixation of a Volar Displaced Salter–Harris III Mallet Fracture in a Pediatric Patient: A Case Report	PMID:40863716	identical DOI

S2.7. Corpus topology and external contextual evidence

The controlling included-source denominator is the 60-row S2 evidence map and its 60-entry included bibliography. The main manuscript reference list also cites methods and external context; it is not a one-to-one included-source count. Jain et al. (NINJA) is external contextual randomized evidence and is outside the 60-source map. It is used only to contextualize nail replacement after uncomplicated pediatric nail-bed repair. Because infected injuries and distal-phalanx fractures requiring fixation were excluded, NINJA remains indirect for Seymour injuries and is not evidence for Seymour fixation decisions.

Immobilization duration was not comprehensively synthesized in this review; therefore, no universal duration is proposed.

S2.8. Population scope and title decision

The revised title uses ‘Children and Adolescents’ because skeletal maturity was not reported consistently across the 60-source map. Pediatric-specific quantitative interpretation required separable pediatric data. Two legacy mixed-age reports with nonseparable or incompletely reported age strata were retained only as contextual phenotype or technique descriptions and were not used for pediatric-specific quantitative inference. The source-level scope constraints below make these boundaries explicit; no retrospective exclusion was performed.

Source	Population fact	Role in the evidence map	Interpretive boundary
10 — Al-Qattan 2001; PMID 11386767	Mixed child, adolescent, and adult case series, n=25; pediatric subset not separable.	Contextual description of Seymour-type morphology and reported management.	Not used for pediatric-specific quantitative inference or treatment comparison.
13 — Zipoli et al. 2010; PMID 20724203	Case series, n=4; child/adult distribution not extractable from the archived abstract.	Contextual mechanism and technique description.	Not used for age-specific quantitative inference; access/reporting limitation remains explicit.
25 — Chen et al. 2018; PMID 29399000	Seventeen adolescents; mean age 15.2 years; physes closed or nearly closed.	Adolescent bony-mallet comparator evidence under the ‘Children and Adolescents’ title.	Does not support a claim that all mapped participants were skeletally immature.
17 — Han et al. 2015; PMID 26279261	Pediatric bony mallet avulsion described without physeal injury.	Age-defined comparator morphology.	Not classified as a physeal Seymour injury and does not alter the denominator.

Source	Population fact	Role in the evidence map	Interpretive boundary
51 — Hosozawa et al. 2025; DOI 10.1016/j.joscr.2024.12.001	Premature physeal closure was reported as an outcome/complication.	Outcome evidence within the pediatric map.	Outcome-related closure is not treated as evidence of baseline maturity or immaturity.

Resolved decision: the title uses ‘Children and Adolescents’ because the map is age/cohort-defined and does not demonstrate skeletal immaturity for every included source. Reported physeal status is charted when available and is not inferred from age alone. Two mixed-age sources with inseparable age strata are retained only as contextual descriptions and are not used for pediatric-specific quantitative inference. No source was retrospectively removed and the n=60 denominator is unchanged.

S2.9. Completed update-candidate decisions outside the historical 60-source map

ID	Title	Eligibility dimensions to assess	Reviewer decisions	Corpus status
W2951549029	Interventions for treating fingertip entrapment injuries in children	Target phenotype; separable pediatric evidence; eligible report type; extractable finding.	FL: EXCLUDE (23 Aug); NL: EXCLUDE (25 Aug); consensus: EXCLUDE.	Outside historical 60; report-assessment exclusion.
W7202014356	Mallet Finger	Publication status; pediatric/adolescent population; target phenotype; eligible report type; extractable evidence.	FL: EXCLUDE (23 Aug); NL: EXCLUDE (25 Aug); consensus: EXCLUDE.	Outside historical 60; report-assessment exclusion.
W7200484546	Effect of a physiotherapeutic intervention in pediatric patients with postpartum plexus brachialis paresis assessed by the Active Movement Scale and Mallet Grading System	Traumatic distal-phalanx concept; publication type; eligibility.	FL: EXCLUDE (23 Aug); NL: EXCLUDE (25 Aug); consensus: EXCLUDE.	Outside historical 60; title/abstract exclusion.

No update candidate is incorporated in the 161-record register, 58-duplicate log, 26 report exclusions, 60-source map, or Figure 1. The separate FL/NL register contains the decisions, reasons, identities, and dates; all three records were excluded and no historical count changed.
